# Activation of Myenteric Glia during Acute Inflammation *In Vitro* and *In Vivo*

**DOI:** 10.1371/journal.pone.0151335

**Published:** 2016-03-10

**Authors:** Corinna Rosenbaum, Martin Alexander Schick, Jakob Wollborn, Andreas Heider, Claus-Jürgen Scholz, Alexander Cecil, Beate Niesler, Johannes Hirrlinger, Heike Walles, Marco Metzger

**Affiliations:** 1 Department of Tissue Engineering and Regenerative Medicine (TERM), University Hospital Wuerzburg, Wuerzburg, Germany; 2 Department of Anaesthesia and Critical Care, University of Wuerzburg, Wuerzburg Germany; 3 Department of Anesthesiology and Intensive Care Medicine, University Medical Center Freiburg, Freiburg, Germany; 4 Translational Centre for Regenerative Medicine, University of Leipzig, Leipzig, Germany; 5 Interdisciplinary Centre for Clinical Research (IZKF), University Hospital Wuerzburg, Wuerzburg, Germany; 6 Department of Bioinformatics, University of Wuerzburg, Wuerzburg, Germany; 7 Department of Human Molecular Genetics and nCounter Core Facility, Institute of Human Genetics, University of Heidelberg, Heidelberg, Germany; 8 Carl-Ludwig-Institute for Physiology, University of Leipzig, Leipzig, Germany; 9 Department of Neurogenetics, Max-Planck-Institute for Experimental Medicine, Goettingen, Germany; 10 Translational Center ‘Regenerative Therapies for Oncology and Musculoskeletal Diseases’ (TZKME), Branch of the Fraunhofer Institute Interfacial Engineering and Biotechnology (IGB), Wuerzburg, Germany; University of California, Los Angeles, UNITED STATES

## Abstract

**Background:**

Enteric glial cells (EGCs) are the main constituent of the enteric nervous system and share similarities with astrocytes from the central nervous system including their reactivity to an inflammatory microenvironment. Previous studies on EGC pathophysiology have specifically focused on mucosal glia activation and its contribution to mucosal inflammatory processes observed in the gut of inflammatory bowel disease (IBD) patients. In contrast knowledge is scarce on intestinal inflammation not locally restricted to the mucosa but systemically affecting the intestine and its effect on the overall EGC network.

**Methods and Results:**

In this study, we analyzed the biological effects of a systemic LPS-induced hyperinflammatory insult on overall EGCs in a rat model *in vivo*, mimicking the clinical situation of systemic inflammation response syndrome (SIRS). Tissues from small and large intestine were removed 4 hours after systemic LPS-injection and analyzed on transcript and protein level. Laser capture microdissection was performed to study plexus-specific gene expression alterations. Upon systemic LPS-injection *in vivo* we observed a rapid and dramatic activation of Glial Fibrillary Acidic Protein (GFAP)-expressing glia on mRNA level, locally restricted to the myenteric plexus. To study the specific role of the GFAP subpopulation, we established flow cytometry-purified primary glial cell cultures from GFAP promotor-driven EGFP reporter mice. After LPS stimulation, we analyzed cytokine secretion and global gene expression profiles, which were finally implemented in a bioinformatic comparative transcriptome analysis. Enriched GFAP+ glial cells cultured as gliospheres secreted increased levels of prominent inflammatory cytokines upon LPS stimulation. Additionally, a shift in myenteric glial gene expression profile was induced that predominantly affected genes associated with immune response.

**Conclusion and Significance:**

Our findings identify the myenteric GFAP-expressing glial subpopulation as particularly susceptible and responsive to acute systemic inflammation of the gut wall and complement knowledge on glial involvement in mucosal inflammation of the intestine.

## Introduction

Enteric glial cells (EGCs) are the most abundant cells within the enteric nervous system (ENS). EGCs are located adjacent to neurons within the enteric ganglia and along interganglionic connectives of the myenteric and submucosal plexus, but also project into the extraganglionic mucosal layer [1–5]. They resemble central nervous system (CNS) astrocytes in their morphology [3,6] and in the expression of shared markers such as the intermediate filament protein glial fibrillary acidic protein (GFAP) [7] and the calcium-binding protein S100 [8]. On a functional level both glial cell types resemble in their reactivity to an inflammatory environment. Like astrocytes [9], EGCs can exert immunomodulatory functions, they can express major histocompatibility complex (MHC) class II molecules [10,11] and secrete inflammatory signaling molecules such as interleukins IL-1β and IL-6 [12,13] as well as other mediators including nerve growth factor (NGF), S-Nitrosoglutathione (GSNO), nitric oxide (NO) and S100B [14]. Inflammation-activated CNS astrocytes are characterized by hypertrophy and proliferation, along with an upregulation of cytoskeletal GFAP [15]. Similarly, under inflammatory bowel disease (IBD) conditions, gliogenesis occurs within the EGC network [16] and prominent alterations in the expression levels of GFAP as well. In ulcerative colitis (UC) patient biopsies, mucosal GFAP expression levels are significantly elevated in inflamed region whereas in Crohn’s disease (CD) patients GFAP expression levels are lower and even significantly reduced in non-inflamed mucosal biopsies compared to healthy controls [17,18]. Additionally, EGCs upregulate expression of intracellular GFAP after experimental acute inflammatory stimulation *in vitro* [19]. Even though this inflammation-induced increase in GFAP expression is well-described, the pathophysiological role of the GFAP-expressing EGC-subpopulation remains largely obscure.

So far, studies on EGC activation and GFAP upregulation have exclusively focused on mucosal glial processes. Mucosal inflammation as in IBD occurs when the finely regulated communication between the host mucosal immune system and the outside microbiota is dysregulated at their contact site, the epithelial barrier. Glial processes in the mucosa have gained attention since they are in intimate association with the epithelial cell layer, form a dense network at the epithelial crypts and release mediators that increase intestinal barrier integrity, potentially beneficial to counteract barrier breakdown [1,20,21,22]. However, the majority of EGCs are located outside the mucosa in the myenteric and submucosal plexus structures. Their activation and contribution to inflammation progression is largely unknown. Recently, it was shown that plexus-originating glial cells continuously invade the lamina propria to form the mucosal glial network, dependent on signals emanating from the gut microbiota [23]. These findings render ganglionated glial cells highly interesting for the study of inflammation-induced alterations.

Furthermore, studies on EGC pathophysiology have previously focused on local intestinal inflammation originating within the mucosa, and little is known about other inflammatory conditions affecting the bowel. One such condition is the systemic inflammation response syndrome (SIRS) or sepsis, a life-threatening complication in clinically ill patients commonly seen after impairment of gut motility due to surgical procedures or trauma [24]. The intestine holds a special position in disease onset and progression, since it is both a source and target of the infection. Local intestinal inflammation, triggered by intestinal manipulation, can lead to release of inflammatory mediators that enter mesenteric lymph and bacterial translocation across the epithelial barrier into the bloodstream, thereby spreading to sites distant from the initial local inflammation [25,26]. The subsequent secondary septic infection can cause multiple organ dysfunction, affecting the entire intestine and other organs.

In the present study, we investigate alterations in the glial network in a lipopolysaccharide (LPS)-induced acute systemic inflammation rat model *in vivo* [27] to test if activation of EGCs known to occur in mucosal inflammation can be observed also in a severe systemic inflammation. Indeed, we found rapid upregulation of *Gfap* gene expression upon intravenous LPS-injection, however specifically confined to the myenteric plexus.

To further characterize this GFAP-expressing myenteric glial subpopulation, we conducted an *in vitro* study with purified primary murine GFAP-EGCs cultured as gliospheres. Microarray and proteomics analysis of LPS-stimulated gliospheres confirmed specific molecular association of myenteric GFAP-glia with immunologic processes. Taken together, our study underlines the pivotal role of myenteric glia during acute gut inflammation and sepsis.

## Materials and Methods

Animal Research was approved by the Ethics Committee of the District of Unterfranken, Wuerzburg, Germany. Approval number: 48/10

### Rat animal model of hyperinflammation

Rats were employed for the hyperinflammation model because the downscaling of the elaborate intensive care setup, usually employed for human patients, was feasible to the size of rats but not of mice. Upon animal care committee approval 10 male Sprague Dawley rats (mean weight: 0.330±0.025 kg) were obtained from Harlan Winkelmann (NM Horst, Netherlands) and kept on a standard diet and water *ad libitum* at 12 h day and night cycles. Hyperinflammation was induced as previously described [27,28]. Briefly, a continuous intensive care set-up was chosen to closely monitor temperature and macrohemodynamic changes. Animals were anaesthetized using isoflurane 1.5–2.5% (v/v) (Forene, Abbott, Wiesbaden, Germany)–nitrous oxide inhalation 50% v/v (Forene, Abbott) and anaesthesia was deepened during the procedure using 0.7% (v/v) isoflurane with a continuous intravenous application of fentanyl and midazolam (Midazolam-ratiopharm, Ratiopharm, Ulm, Germany) discontinuing the nitrous oxide.

Rats were randomized into a control and a LPS-treated group, n = 5 each. Control animals received a 0.5 ml intravenous bolus of NaCl as placebo, whereas Lipopolysaccharide (LPS; E. coli O111:B4, Sigma Aldrich, Deisenhofen, Germany; potency 3 EU/ng) was applied intravenously at a concentration of 2.5 mg/kg in the LPS group. After 3.5 h, laparotomy was performed and 4 h after the LPS-injection animals were sacrificed via overdose of isoflurane. Gut tissue samples of 1–2 cm length were taken from duodenal, ileal and proximal colonic parts. For histochemical analyses samples from each animal and intestinal region were embedded in OCT compound (Tissue Tek; Sakura Finetek, Tokyo, Japan), frozen on dry ice and stored at -80°C. For gene expression analyses samples were submersed with RNAlater^™^ (Qiagen, Hilden, Germany) overnight at 4°C, afterwards removed from the reagent and transferred to -80°C until further use.

### Immunohistochemistry

Immunohistochemistry was performed on 12 μm cryosections, small intestinal longitudinal muscle-myenteric plexus (LMMP) strips, and gliospheres. Samples were fixed with 4% paraformaldehyde (PFA, AppliChem, Darmstadt, Germany). Blocking of unspecific binding sites was achieved by incubating sections with 5% donkey serum (Sigma Aldrich, Germany) in 0.2% Triton-X 100 (Carl Roth, Karlsruhe, Germany). The following primary antibodies were diluted in Lab Vision^™^ Antibody Diluent OP Quanto (Thermo Scientific, Dreieich, Germany) and incubated overnight at 4°C: rabbit anti-GFAP (1:600; Dako, Hamburg, Germany), mouse anti-HuC/D (clone 16A11, 1:100; Invitrogen, Germany), guinea pig anti-Sox10 (1:700, kindly provided by Prof. Wegner, Institute of Biochemistry, University Erlangen [29]), rabbit anti-p75 neurotrophin receptor (1:250, Promega, Mannheim, Germany), mouse anti-alpha smooth muscle Actin (clone 1A4, 1:100, Abcam, Cambridge, United Kingdom) and rabbit anti-Vimentin (clone EPR3776, 1:100, Abcam).

After washing, samples were incubated for 1 hour at room temperature with secondary antibodies. Following washing, sections were mounted with Mowiol/1,4-diazabicyclo[2.2.2]octane (DABCO) containing 0.1% 4',6-diamidino-2-phenylindole (DAPI, Carl Roth) and analyzed using an inverse fluorescence microscope (Keyence, BZ-7000, Tokyo, Japan and Zeiss Axiovert V.1, Jena, Germany).

For quantification of stained nuclei 10 representative images with 20x magnification were taken per intestinal region (duodenum, ileum, proximal colon) of each sample (n = 5 per group), counted in a blinded procedure and averaged. For pixel area measurements images were taken accordingly. Fractional area measurements were performed of the intramuscular GFAP-staining with ImageJ software (v1.38; National Institutes of Health, Bethesda, USA.) with default settings.

### RNA isolation and quantitative RT-PCR

Total RNA was isolated using the RNeasy Micro Kit according to the manufacturer’s instructions (Qiagen). 0.5 μg of total RNA was processed for reverse transcription using iScript^™^ cDNA Synthesis Kit, following the supplier’s protocol (Biorad, Muenchen, Germany). Quantitative real-time PCR was performed in accordance to the MIQE-guidelines [30] with the CFX96 Touch^™^ Real-Time PCR Detection System (Biorad) using the SsoFast^™^ EvaGreen^®^ Supermix (Biorad). Exon junction-spanning primer pairs were designed with Primer-BLAST Software [31] (S1 Table). Quantitative real time-PCR was performed in duplicate with 0.4 pmol/μl of each primer and 0.5 μg cDNA at an annealing temperature of 60°C. Gene expression data were normalized to the reference genes β-actin (Actb) and 18S rRNA following the 2(-Delta Delta C(T)) method described by Vandesompele [32] using the CFX Manager software (Biorad). The quality of amplified PCR products was proven by melting curve analysis and agarose gel electrophoresis.

### Laser capture microdissection

Laser capture microdissection (LCM) was performed on 12 μm cryosections mounted on UV-light pretreated PEN-membrane covered slides (Carl Zeiss, Jena, Germany). Tissue sections were shortly air-dried and stored at -80°C for LCM. Prior to LCM, cryosections were thawed and stained with RNase-free 1% (w/v) cresyl violet acetate solution (Sigma) according to Carl Zeiss MicroImaging PALM protocols. LCM was performed with the PALM MicroBeam System (Carl Zeiss MicroImaging, Bernried, Germany) according to the manufacturer’s instructions. Captured tissue segments were collected in AdhesiveCaps (Carl Zeiss) until a total tissue area of on average 2.1x10^6^ μm^2^ for myenteric and submucosal structures and of 2.8x10^6^ μm^2^ for mucosal segments was excised. Segments were excised as directly adjacent oval shapes, thereby the enteric myenteric and submucosal regions of the cryosections were collected. Prior to RNA isolation lysis buffer was added and AdhesiveCaps were incubated upside down for 30 min at room temperature. RNA quality from randomized representative samples was assessed in preliminary experiments using the Experion RNA HighSens Assay (Biorad) and yielded good results in both the muscular and mucosal samples (average RIN 7.4).

### Isolation and culture of GFAP-expressing enteric glia

Enteric glial cultures were propagated from FVB/hGFAP-EGFP transgenic mice expressing green fluorescent protein under the control of the human GFAP-promotor [33]. At approximately postnatal day 7, all mice of the same litter were sacrificed by decapitation, small intestines removed, screened for GFP-positivity, pooled and further processed as described before [34]. Cells were resuspended in Dulbecco’s modified Eagle medium (DMEM)/F-12 medium supplemented with Glutamax (Life Technologies, Darmstadt, Germany), penicillin (100 U mL^-1^; PAA, Coelbe, Germany), streptomycin (100 mg mL^-1^; PAA), B27 without vitamin A (1:50; Invitrogen), N2 (1:100; Invitrogen), sorted for GFP by flow cytometry and seeded onto culture dishes coated with poly-ornithine (0.01%), fibronectin and laminin (both 1 μg cm^-2^; Sigma-Aldrich). Epidermal growth factor (EGF; 40 ng mL^-1^; Peprotech, Hamburg, Germany) and basic fibroblast growth factor (bFGF; 40 ng mL^-1^; Peprotech) were added daily and cells cultured under hypoxic conditions (2% O_2_) in a humidified incubator at 37°C. Cell culture medium was changed by 50% every 2–3 days.

For LPS-stimulation experiments, cells were passaged after 2 weeks and seeded on uncoated plastic dishes to promote spheroid formation. Cells from each animal preparation (n = 3) were split into two six well dishes, one stimulated with LPS (100 μg mL^-1^, from E. coli O26:B6, Sigma Aldrich, potency 3 EU/ng). After 48 h, cell culture supernatants were collected for cytokine measurements and cells processed for total RNA isolation.

### Microarray analysis

Total RNA from gliospheres was extracted and RNA integrity (RIN > 7) assessed using the Bioanalyzer technology (Agilent Technologies, Waldbronn, Germany) and further processed on Illumina microarrays according to the manufacturer’s instructions (MouseRef-8v2.0 Expression BeadChip, Illumina, San Diego, USA). Data analysis was performed at the IZKF core facility and Dept. of Bioinformatics of University Wuerzburg. Briefly, raw data was background-corrected (normexp method) and normalized (VSN method); microarray probes referring to unexpressed genes (detection p-value > 0.05 in all samples), known bad assay performance or with no assigned genes were removed from the dataset. Moderated t-tests were used for detection of differentially expressed genes. Analyses were performed in the R environment (Version 3.3.1) [35] with Bioconductor extension packages “vsn”, “made4”, “limma” and “illuminaMousev1p1.db”. Enrichment analyses were performed with DAVID [36,37].

To visualize the differentially expressed genes and their association with Gene Ontology (GO) terms [38,39], a color-coded Voronoi diagram was created [40]. We selected GO annotation terms of the same high hierarchical level to achieve accumulation of a limited set of terms. The calculation of the Delaunay triangulation and the subsequent calculation of Voronoi tessellation was done in R by application of the deldir package (Version 0.1–9) [41].

Data have been deposited in NCBI's Gene Expression Omnibus [42] and are accessible through GEO Series accession number GSE78015 (https://www.ncbi.nlm.nih.gov/geo/query/acc.cgi?acc=GSE78015).

### Comparative transcriptome analysis

The global transcriptional datasets generated in this study were compared with data from previous studies, deposited in public databases (NCBI Gene Expression Omnibus, GEO; S2 Table) [42]. R/Bioconductor and the software package “virtualArray” were used to combine all data into one dataset (virtual array) [35,43]. The resulting new array was exported and analyzed in TM4 MeV [44] and Archaeopteryx [45], which was further used to employ hierarchical clustering analysis to show similarities or differences to cells of related identity or other cells of interest [46].

### nCounter analysis

The nCounter technology (NanoString^®^ Technologies, Seattle, USA) was employed to validate microarray data as described before [47]. A customized CodeSet was designed consisting of 60 genes identified in the microarray dataset as particularly interesting candidates, including 7 reference genes. The CodeSet probes were designed to bind at the same nucleotide position as the Illumina probes used in the microarray assay (S3 Table). In order to yield sufficient amounts of RNA, we pooled intestines of 5 separate litters of transgenic hGFAP-EGFP animals and measured in three technical replicates. Data acquisition and normalization was carried out using the nSolver analysis software version 2.5 (NanoString^®^ Technologies).

### Cytokine 20-Plex Bead Array

Cytokine and chemokine contents of gliosphere culture supernatants were quantified using the multiplexed protein profiling Luminex^®^-method following the manufacturer’s protocol (Cytokine Mouse 20-Plex Panel; Invitrogen, Karlsruhe, Germany). Analyses were done in triplicates as previously described [48,49] (Luminex Corporation, Austin, USA) employing the StarStation software (ACS, Sheffield, UK).

### Statistical analyses

Statistical analysis was performed with the nonparametric Mann-Whitney-U-Test or, if the criterion of normal distribution was met, with univariate ANOVA. All statistical tests were performed using SPSS (IBM). Results are given as mean ± standard deviation. The level of statistical significance was set at p<0.05, indicated with asterisk (*). Grading in significance is indicated as follows: *p<0.05, **p<0.01, ***p<0.001. A p-value between 0.05 and 0.10 was considered as moderate evidence for statistical significance and biological relevance and indicated with a hash (^#^).

## Results

### Gene expression analysis of enteric glia in a hyperinflammation rat model *in vivo*

**Fig 1:**
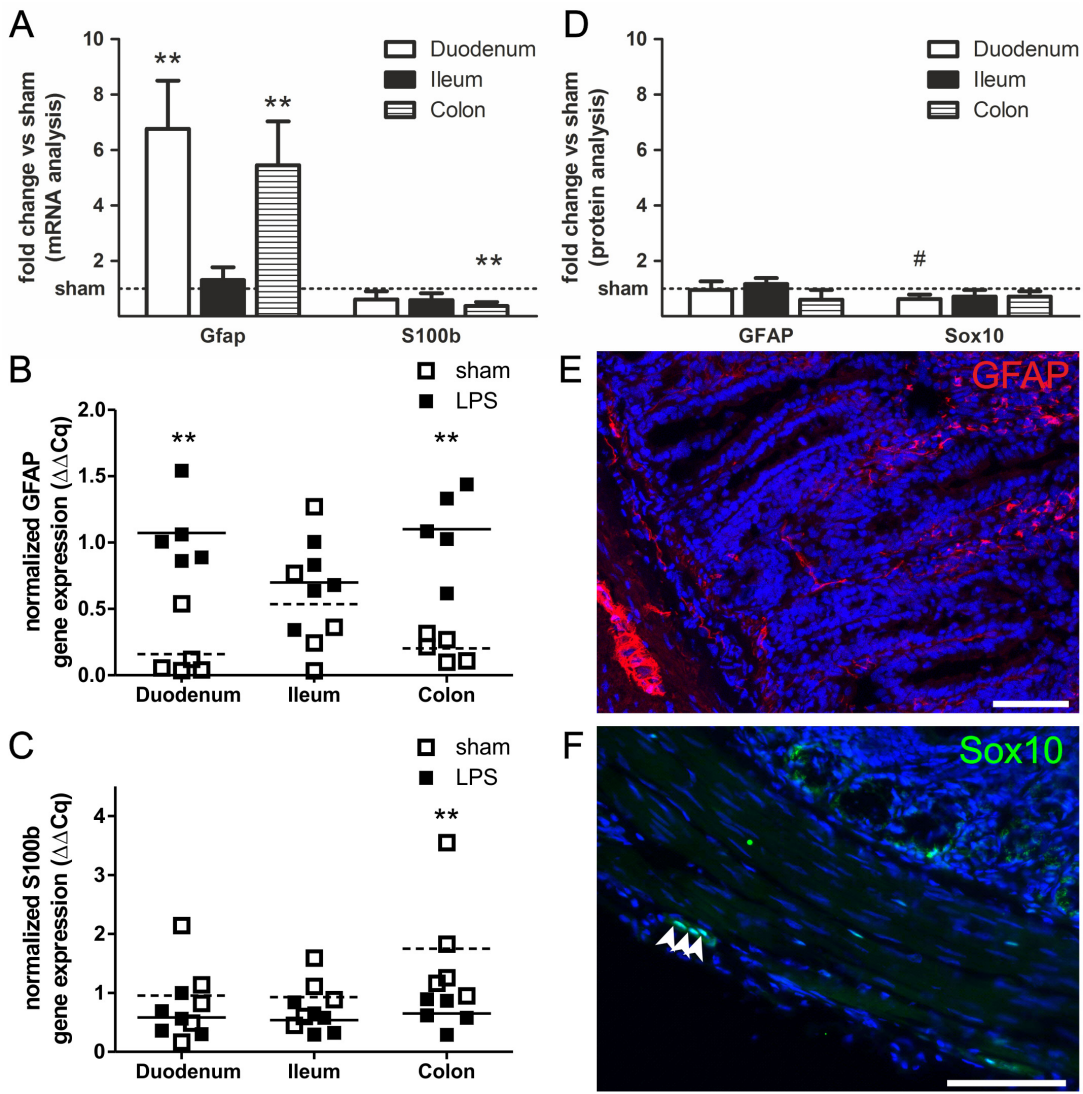
*Gfap* gene expression upregulation in hyperinflamed intestinal rat tissue. (A) Quantitative PCR analysis of glial marker expression in whole intestinal wall preparations from duodenal, ileal and colonic segments shows that *Gfap* gene expression but not *S100b* expression is upregulated in hyperinflamed gut tissue. We found increases in *Gfap* expression in duodenal segments by 6.8-fold and in colonic segments by 5.5-fold with a concurrent decrease of *S100b* expression to the 0.4-fold. In ileal samples no significant changes were observed. Absolute normalized gene expression of Gfap (B) and S100b (C) normalized to the reference genes (ΔΔCq). Means of LPS-treated samples (filled symbols) are indicated with lines, means of sham-treated samples (empty symbols) are indicated with dashed lines. (D) Quantification of immunohistochemical stainings for intramuscular GFAP (E, representative duodenal staining) and the glial transcription factor Sox10 (F, representative duodenal staining). 4 h after LPS injection we did not observe any significant alterations on protein level. Counts of Sox10-positive nuclei in the myenteric plexus show a tendency to be reduced upon LPS treatment in the duodenum, on average we counted 3.6 Sox10-positive nuclei per image in LPS-injected animals and 5.8 nuclei in sham-treated controls. Results (mean ± standard deviation) are given as fold change relative to sham group set to 1, as indicated by the horizontal line, n = 5, ^#^ p < 0.10, ** p < 0.01, scale = 100 μm.

The effects of a systemic inflammatory insult on the EGC network were analyzed in an *in vivo* LPS-mediated hyperinflammation rat model. Glial marker expression was quantified on mRNA and protein level in duodenal, ileal and colonic tissue segments. By quantitative PCR analysis we found significantly increased levels of *Gfap* gene expression in the duodenal (6.8±1.7-fold; p < 0.01) and colonic (5.5±1.6-fold; p < 0.01) segments of whole intestinal wall preparations 4 h after intravenous LPS injection compared to sham-treated controls (Fig 1A–1C). This increase did not coincide with an overall glial marker expression increase, since *S100b* expression in fact decreased after LPS treatment to 0.6-fold in duodenal and ileal, and to 0.4±0.1-fold (p < 0.01) in colonic segments. In contrast to the duodenum and colon, *Gfap* expression in ileal segments showed no significant alteration, however the sham group already reached relative expression values close to those observed in LPS-treated duodenal and colonic samples.

**Fig 2:**
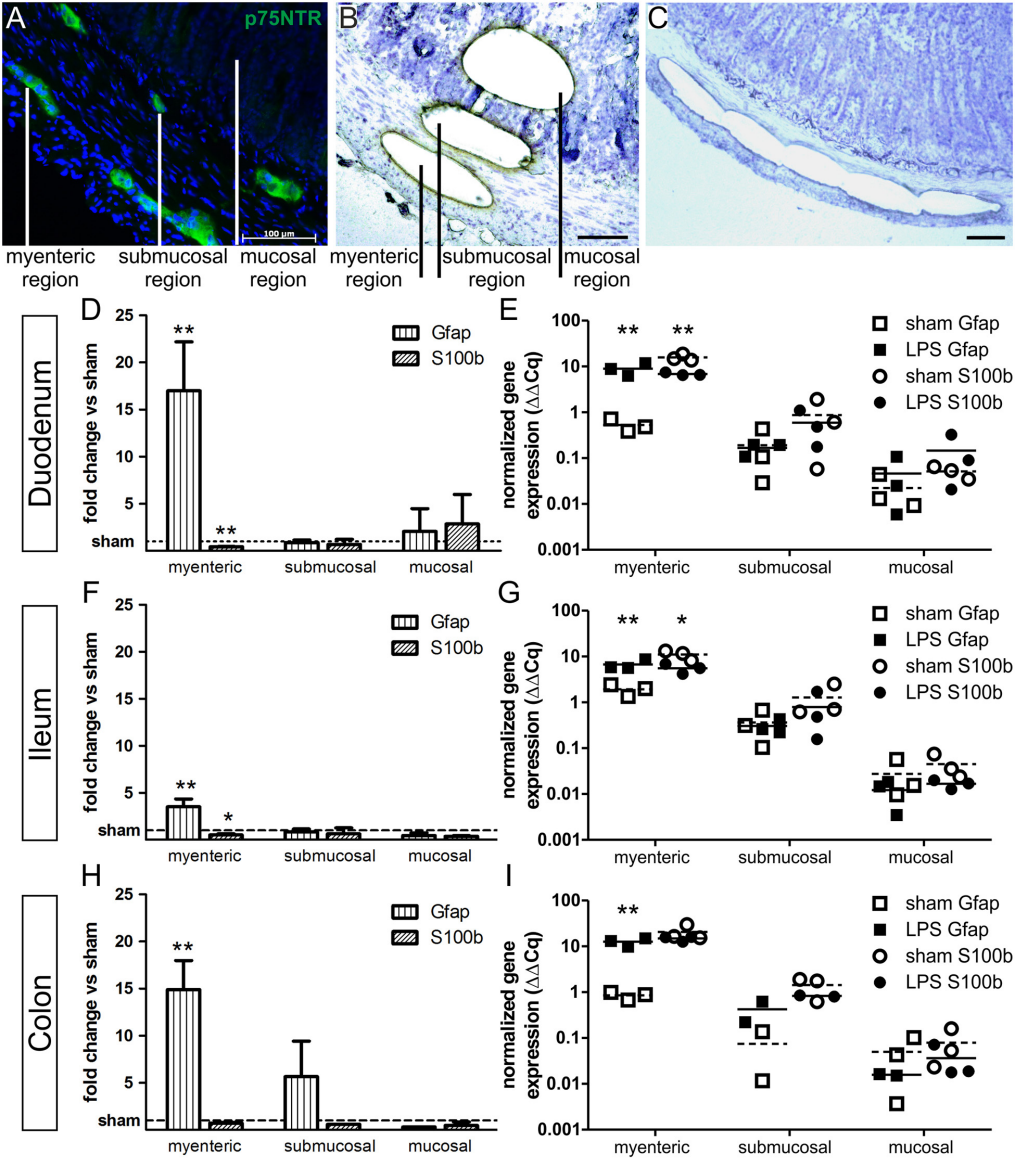
LPS-induced *Gfap* and *S100b* gene expression alterations are confined to the myenteric plexus. (A) Immunohistochemical staining of p75 neurotrophin receptor (p75NTR) indicating the position of myenteric and submucosal plexus structures within the tissue. (B-C) Cresylviolet-stained tissue section showing exemplary myenteric, submucosal and mucosal tissue segments cut and catapulted for mRNA transcript level analysis (B) and sequential excision of segments, here shown for the myenteric region (C). (D, F, H) Quantitative PCR analysis of laser-dissected myenteric, submucosal and mucosal tissue segments of duodenal (D), ileal (F) and colonic (H) samples. In all intestinal regions the significant upregulation of *Gfap* up to the 17.2±5.2 fold change in the duodenum and downregulation of *S100b* gene expression is confined to the myenteric plexus. Results (mean ± standard deviation) are given as fold change relative to sham group set to 1. (E, G, I) Absolute normalized gene expression of Gfap (sqares) and S100b (circles) in duodenal (E), ileal (G) and colonic (I) segments, plotted on a logarithmic scale. Means of LPS-treated samples (filled symbols) are indicated with lines, means of sham-treated samples (empty symbols) are indicated with dashed lines. n = 3, *p<0.05, **p<0.01, scale = 100 μm.

Next, we aimed to analyze the region-specificity of LPS-responsive glia in our model. Thus, we excised myenteric, submucosal and mucosal tissue segments via laser capture microdissection (Fig 2A) and performed subsequent qPCR analysis. Interestingly, the LPS-group revealed a highly significant mRNA increase in the myenteric plexus of duodenal samples by 17.2±5.2-fold (Fig 2B, p < 0.01), of ileal *Gfap* level by 3.5±0.8-fold (p < 0.01, Fig 2C) and of colonic samples by 15.0±3.1-fold (Fig 2D, p < 0.01). As described before, we observed a concurrent decrease in *S100b* expression, reaching significant values in the small intestine to the 0.4-fold in the duodenum (p < 0.01) and to the 0.5-fold in the ileum (p < 0.05). Noteworthy, no significant effects on *Gfap* or *S100b* expression were detected within the submucosal and whole mucosal regions.

Despite the fact that significant protein changes after only 4 h of treatment might not be expectable due to the delay of translation we included estimation of protein expression levels, too. We quantified GFAP+ and Sox10+ cells within the muscular layer/myenteric plexus on histological sections after immunohistochemistry (Fig 1B–1D). The cytoplasmic GFAP (Fig 1C) was quantified by pixel measurements and Sox10+ cells were determined by counting the Sox10+/DAPI+ co-labeled cell nuclei (Fig 1D). We observed a statistical tendency for LPS-induced reduction of Sox10+ glia by 38% in the duodenum (p < 0.10), in total numbers a reduction from on average 5.8 Sox10+ nuclei counted per image to 3.6 Sox10+ nuclei. The intramuscular GFAP quantification showed no significant alterations 4 h after LPS injection.

In analogy to the glial cell analysis, we also assessed neuronal mRNA transcript and protein levels upon LPS-treatment (S1 Fig). However, neither pan-neuronal markers (*Uchl1/PGP 9*.*5*, HuC/D) nor neuronal subtype markers such as for the cholinergic (*Chat*) and nitrinergic (*Nos1*) subpopulations showed statistically significant alterations upon LPS stimulation. Similar to the glial Sox10 protein analysis we merely observed a statistical trend for decreased neuronal cell number in the small intestine by 30% at most (S1B Fig). To summarize, 4 h after LPS injection the overall neuronal and glial populations showed a tendency for reduced cell numbers, but the myenteric glial subpopulation showed a rapid activation and *Gfap* upregulation was only detected on mRNA level within the experimental time frame.

### Gene expression analysis of GFAP-glia after LPS-stimulation *in vitro*

**Fig 3:**
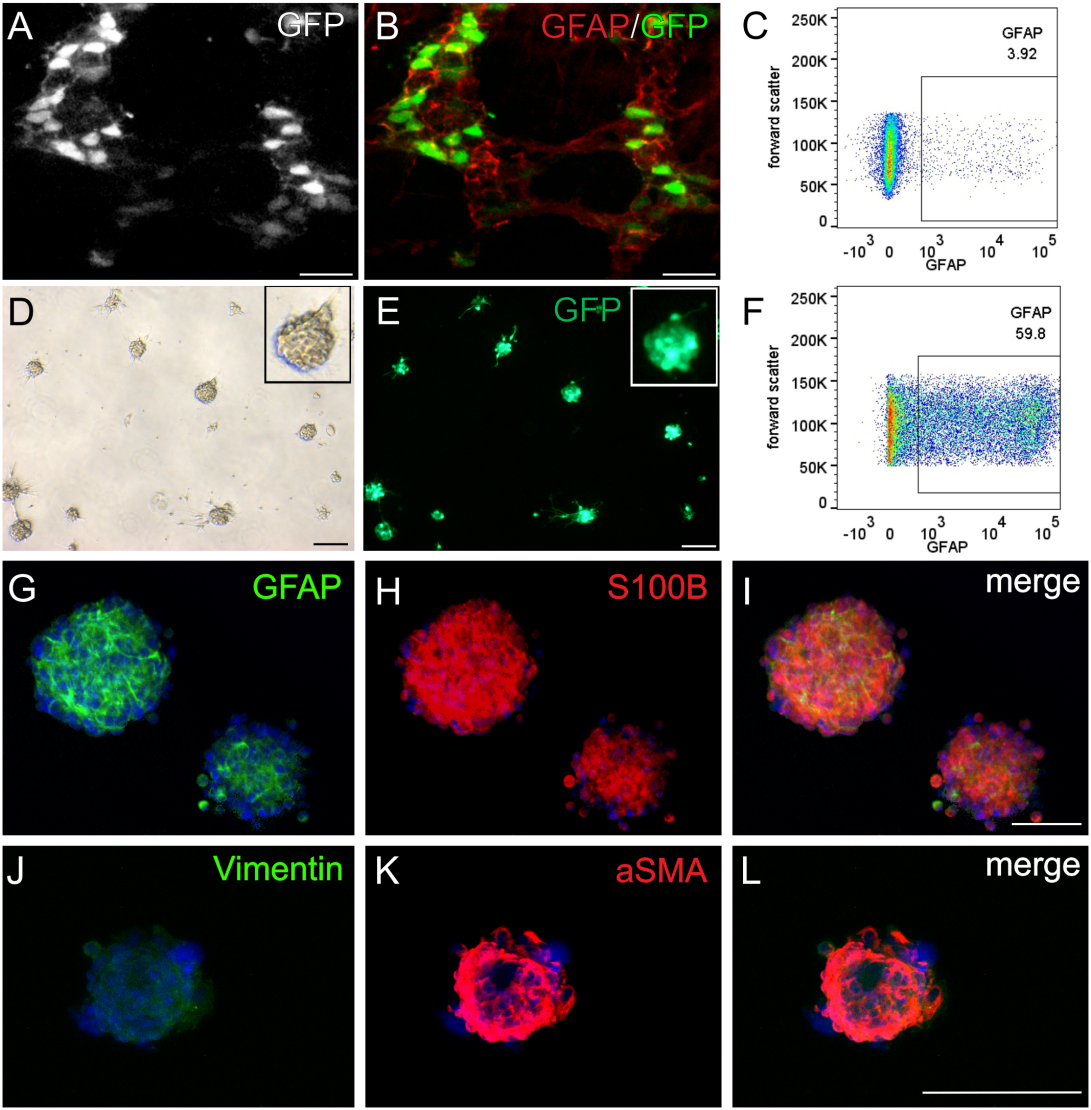
GFAP-expressing glia sorted for EGFP reporter via flow cytometry and expanded in culture. (A) Endogenous GFP fluorescence and (B) overlay with GFAP antibody staining in LMMP strips from transgenic postnatal day 7 mice visualizes that only cells within the plexus structures express GFP in varying intensities. (C) Flow-cytometry analysis of the muscle tissue digest; the gated GFAP-GFP-positive population was sorted and cultured *in vitro* to propagate gliospheres. (D) Brightfield and (E) fluorescence microscopy of gliospheres cultured for 2 weeks *in vitro* shows the formation of gliospheres with a majority of cells expressing GFP. (F) Flow-cytometry analysis of glial culture 2 weeks after initial cell sorting reveals a strong GFP-positivity of about 60% of the total cell population. Immunohistochemical stainings show that the major proportion within gliospheres consists of glial cells positive for GFAP (G) and S100B (H) but spheres also contain cells expressing alpha smooth muscle actin (αSMA, Fig 3K) but are devoid of strongly vimentin-positive cells (J). Scale = 100 μm.

In addition to the *in vivo* study we further investigated specific functions of the GFAP-expressing glial subpopulation in a prolonged acute inflammatory microenvironment *in vitro*. GFAP-glia were isolated from LMMP-preparations from hGFAP-EGFP transgenic mice by flow cytometry (Fig 3A–3C) and propagated as free-floating primary ‘gliospheres’ from the GFAP+ population (Fig 3D–3F). After two weeks in culture, 60% of the cells within the gliospheres were still strongly GFP-positive as confirmed by flow-cytometry (Fig 3F) and spheres stained positive for GFAP (Fig 3G) and S100B (Fig 3H). Noteworthy, alpha smooth muscle actin-positive (Fig 3K) but no prominent vimentin-positive (Fig 3J) cells were detected.

**Fig 4:**
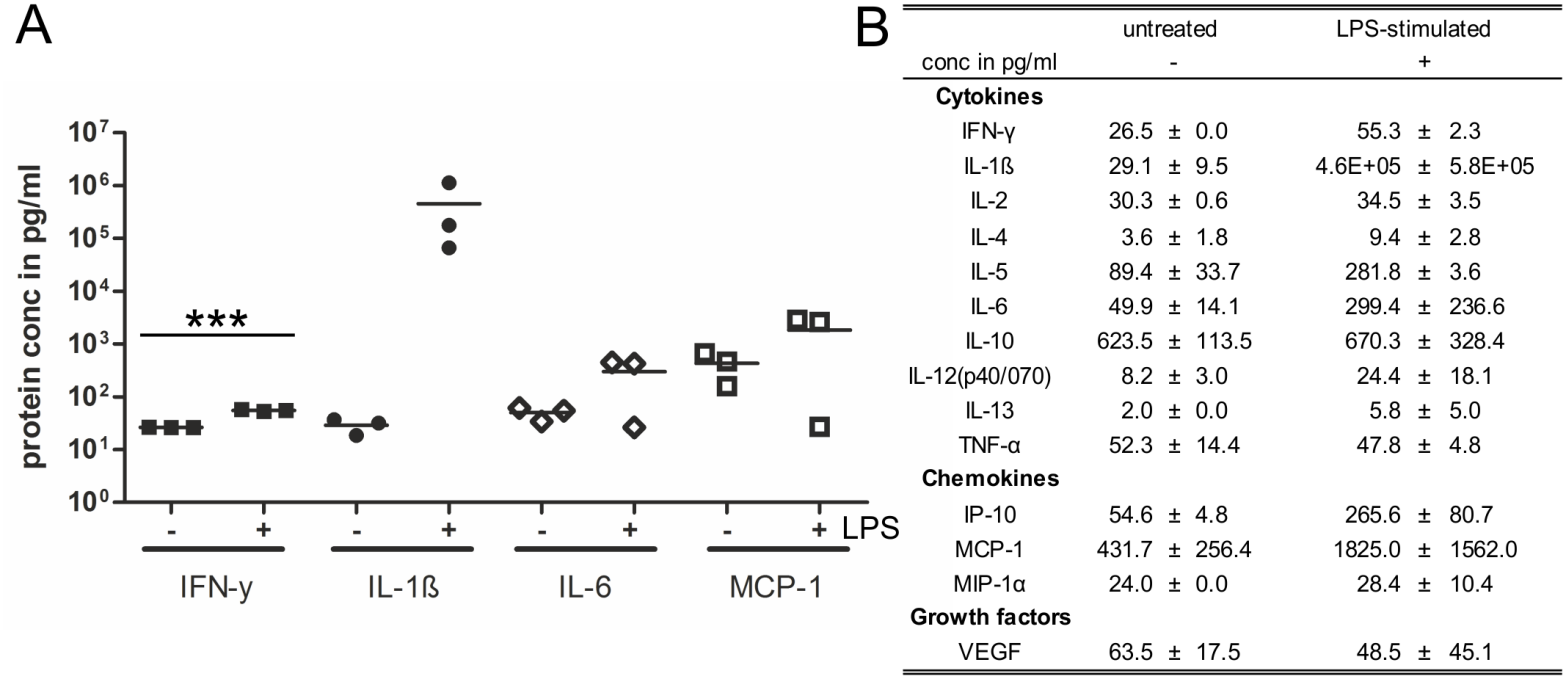
Signaling molecules secreted by GFAP-expressing glia. (A) Secretion of selected pro-inflammatory signaling molecules by untreated (-) and LPS-challenged (+) gliospheres. Each data point represents an independent experiment, n = 3, ***p<0.001. (B) Tabular summary of all signaling molecules measured.

Gliospheres were stimulated with LPS for 48 h and cell culture supernatants were analyzed for secreted cytokines, chemokines and growth factors by multiplex protein profiling (Fig 4). Thus, we detected elevated levels of several pro-inflammatory cytokines including interferon (IFN)-γ, interleukin (IL)-1ß, IL-6 and the monocyte chemoattractant protein (MCP)-1. Only IFN-γ increase reached high statistical significance, the other cytokines showed fluctuating protein concentrations in the independent experiments but an overall increase in secreted levels after LPS-stimulation. Other pro-inflammatory cytokines showed no increased secretion such as IL-2, IL-12 and tumor necrosis factor (TNF)-α. Interestingly, we also detected elevated levels of several anti-inflammatory cytokines including IL-4 and IL-5 suggesting a complex regulatory role of glia.

**Fig 5:**
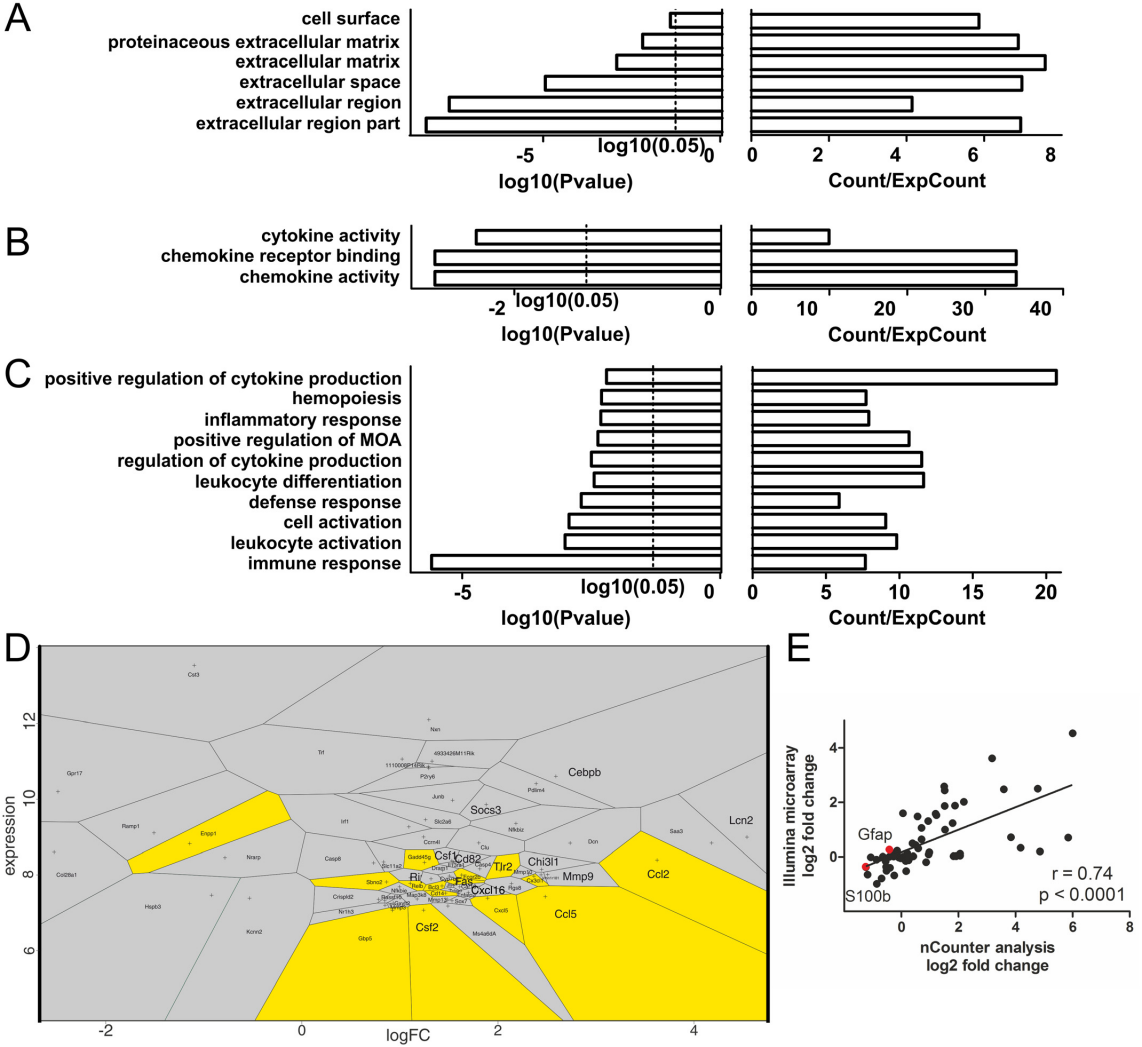
Significantly upregulated glial genes upon LPS stimulation are enriched in immunologic processes. (A-C) Bar charts displaying significantly enriched annotations of differentially expressed genes referring to cellular compartments (A), molecular functions (B) and biological processes (C). The level of significance is given as logarithm (log10) of the p-value calculated using the Benjamini correction. Enrichment is given as the count of genes associated to the respective term in the dataset relative to the expected count (Count/ExpCount). (D) Voronoi diagram displaying each significantly differentially expressed gene. Genes are plotted based on their absolute expression level and the relative expression change in response to LPS treatment given as fold change. The plotting area is segmented and a field is assigned to each gene, its size represents the unique character of the expression parameters. Yellow coloring indicates association of a gene product to the GO-term “immune response”, which is enriched with the highest significance. Genes associated to this term include some of those with the highest fold-change increase upon LPS-stimulation. Increased labeling font size indicates validation of expression with the nCounter technology. (E) Spearman correlation of 60 genes analyzed with the Illumina microarray and the Nanostring nCounter technology. The Spearman coefficient of 0.74 shows highly significant correlation (p<0.0001) of the results obtained with both technologies. Data points for the glial marker genes *Gfap* and *S100b* are highlighted in red.

In the next step, we isolated total RNA from all samples and performed a whole transcriptome analysis to detect overall LPS-induced modifications in gene expression profiles. Thus, we identified 70 differentially expressed genes upon LPS-stimulation (S4 Table). Genes were analyzed by annotations of the representative gene products according to biological functions in the Gene Ontology (GO) database. From this bioinformatics resource significantly over-represented annotations of the gene products to GO-terms were derived, describing their cellular compartment localization, molecular function and involvement in biological processes (Fig 5, S5 Table). For the cellular localization, we found the significantly differentially expressed genes annotated to terms describing the extracellular space (Fig 5A). Respectively, for the molecular function we found over-represented associations to signaling processes such as cytokine and chemokine activity (Fig 5B). For the biological processes the LPS-affected genes were predominantly annotated to immunologic processes, with the GO-term “immune response” showing the most significant enrichment (enrichment by 7.7, p<0.001) (Fig 5C). However, the mere enrichment of the annotation does not imply the degree of differential expression of the respective gene. To visualize the expression parameters of the genes associated to the “immune response”-term, a color-coded voronoi diagram was created (Fig 5D). This diagram shows that the “immune response”-associated genes includes those genes with the highest fold-change increase in the dataset, such as chemokine (C-C motif) ligand 2 and 5 (Ccl2 and Ccl5).

For validation of our microarray data we chose the Nanostring nCounter assay and selected 60 genes, of which 14 ranked in the significantly differentially expressed gene set. Correlating the relative expression level changes upon LPS stimulation measured with both methods revealed a high significance with a Spearman coefficient of 0.74 (Fig 5E, P < 0.0001).

Worth mentioning, neither *Gfap* nor *S100b* transcript levels did change significantly upon LPS-stimulation in our analysis suggesting that the upregulation observed *in vivo* is mainly caused by an initially GFAP-negative cell population (highlighted in Fig 5E).

**Fig 6:**
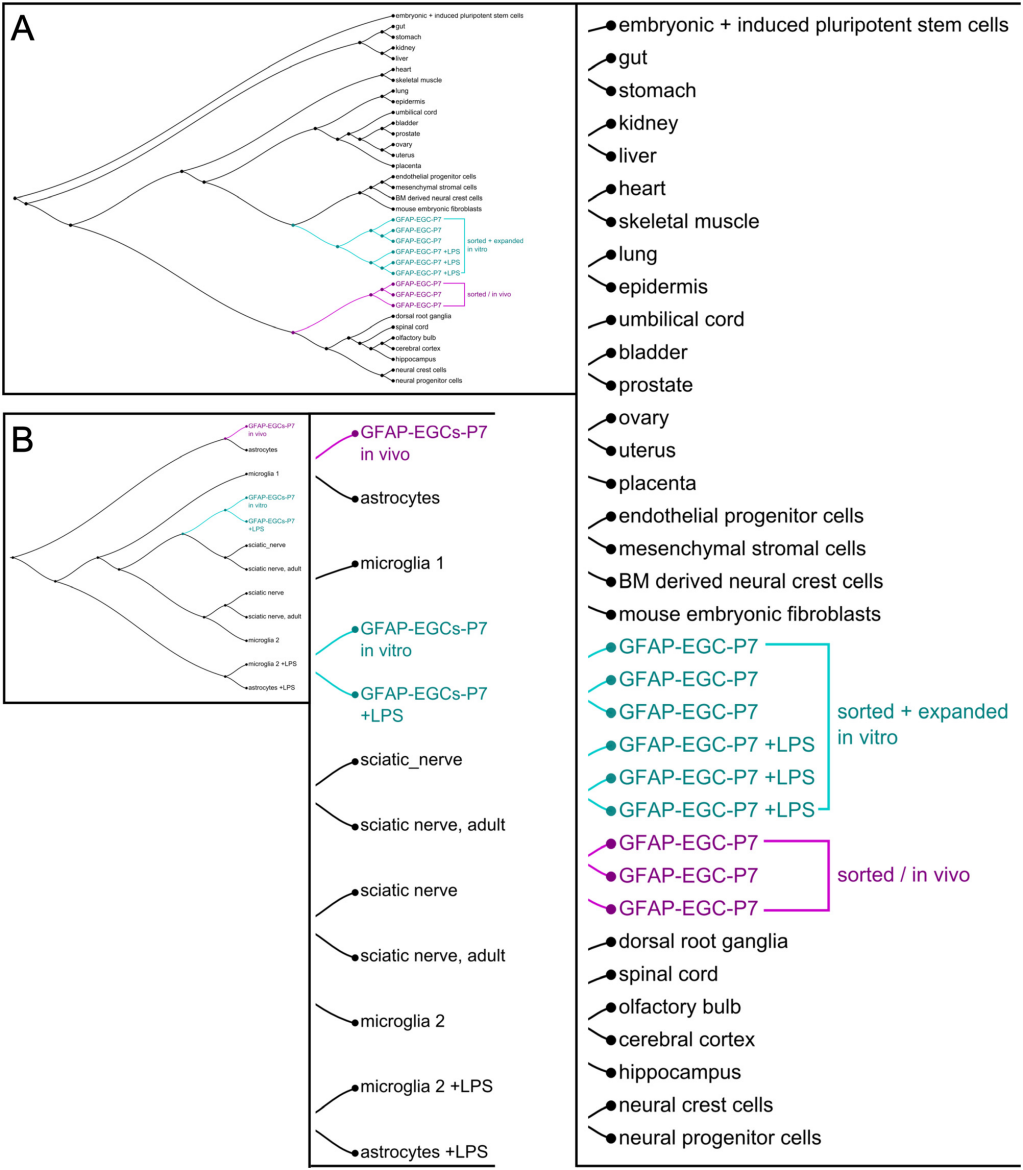
Comparative meta-analyses of multiple GSE datasets reveal genetic similarities among different tissue. (A) and (B) display complete dendrograms, the length along the line connecting two datasets indicates the degree of similarity; the shorter the distance of connecting lines, the more similar these datasets are. For better legibility the list of datasets are enlarged right of the dendrogram overview. (A) Comparative analysis of gene expression profiles of GFAP-expressing glia and profiles of different tissues/cell types reveals a high similarity of GFAP-glia *in vivo* to CNS and PNS nerve cell types and a much lower similarity to GFAP-glia expanded *in vitro*. Gene expression profiles of the latter show similarities to mesenchymal cell types. (B) Hierarchical clustering of glial-specific GSE datasets to show similarities of enteric GFAP-glia to CNS and PNS glia including LPS-challenged cells. Comparative analysis reveals high similarity of GFAP-glia *in vivo* to astrocytes *in vivo*. If cultured *in vitro*, the GFAP-glia genetic profile has higher similarity to PNS sciatic nerve cells and CNS microglia. LPS stimulation leads to severe alterations in gene expression profiles of CNS astrocytes and microglia, which seems not the case for EGCs.

Finally, we extended our analysis to compare the transcriptome data with different tissues and cell types published in the Gene Expression Omnibus (GEO) database [42]. This comparative meta-analysis (Fig 6A) showed that directly sorted intestinal GFAP-glia cluster together with central and peripheral nerve cell types and differ from those GFAP-glia expanded *in vitro* for several weeks. The latter have a much closer transcriptomic resemblance to mesenchymal cell types. Another interesting aspect is that a defined comparison of glial-specific data sets (Fig 6B) revealed the high transcriptomic similarity between enteric GFAP-glia *in vivo* and CNS astrocytes expanded *in vitro*. In contrast, enteric GFAP-glia expanded *in vitro* showed greater resemblance to cells isolated from peripheral sciatic nerves. A further intriguing aspect of this analysis is that LPS-stimulation of astrocytes and microglia has a profound impact on their transcriptomic profile, whereas the impact on enteric GFAP-glia seems relatively moderate.

## Discussion

Enteric glia are known to resemble CNS astrocytes in their reactivity to an inflammatory environment. However, such findings have originated mostly from studies on local mucosal inflammation, either from a chronic disease manifestation as in IBD patients or from acute experimental mucosal manipulation. Little is known about the responsiveness of glia outside the mucosa in the ganglionated structures, which constitute the majority of the EGC network. To study the early response of the entire EGC network to intestinal inflammation not limited to the mucosa, we performed an acute systemic hyperinflammation study *in vivo* induced by intravenous injection of LPS, mimicking the pathological situation observed in postoperative SIRS.

4 h after induction of a systemic inflammation by intravenous LPS-injection we analyzed duodenal, ileal and colonic tissue segments for glial marker expression. We found a tremendous upregulation of *Gfap* mRNA transcript levels. Interestingly, as revealed by LCM, *Gfap* expression regulation was confined to the muscular layer harboring the myenteric plexus. Myenteric glia have recently gained attention in the context of inflammation-induced impairment of gastrointestinal (GI) motility [26]. Stoffels *et al*. identified interleukin-1 receptor (Il-1R1) signaling as essential for surgery-induced inflammation and resulting impaired contractility. The group identified GFAP-expressing myenteric glia as the predominant IL-1R1-expressing resident intestinal cell type, highlighting the potential of GFAP-glia to shape intestinal inflammation. Our findings support this concept of myenteric GFAP-glia as fast-responding, reactive cell types in acute inflammation *in vivo*. Even though a differential susceptibility of EGCs in different plexus structures is an appealing interpretation of our data, it is also plausible that intravenous LPS-injection led to a differing distribution of LPS within the tissue and hence a differing activation of EGCs.

Interestingly, we observed different manifestations of LPS-dependent myenteric *Gfap* expression upregulation along the intestinal proximal-distal axis, being less pronounced in ileal samples. This resulted from already high ileal expression levels in the sham group, which ranged close to those in the LPS-treated duodenal and colonic samples and suggests further factors affecting *Gfap* expression levels. Indeed, our surgical procedure offers a potential explanation: Besides LPS-injection we performed laparotomy and the GI tract was partially exposed. Mild manipulation of the intestine following laparotomy can already be sufficient to induce inflammatory reactions and impaired motility as compared to laparotomy alone [50,51]. Therefore, it is possible that the surgical procedure itself also contributes to an altered *Gfap* expression independent of LPS-stimulation. We consider this “two-hit”-character of our hyperinflammation model to mimic the clinical situation well, since patients become septic usually following a first hit, such as an ileus after severe surgery. Support for a potentially differing susceptibility of ileal GFAP-EGC to external stimuli come from animal models, in which immune-mediated ablation of GFAP-EGCs led to fatal inflammation specifically of the ileum, not the duodenum or proximal colon [52]. Furthermore, different intestinal segments along the proximal-distal axis as well as in different plexus layers vary in glial subtype composition and density [29]. Correlating the plexus composition to the physiological responsiveness of the tissue area will be highly interesting aspect of EGC physiology.

The varying degrees of *Gfap* expression increase we observed were accompanied by a significant decrease in *S100b* gene expression levels in duodenal and ileal myenteric samples as well as colonic whole gut wall samples. Previously, the expression and secretion of the glial-specific enteric S100B has been described to correlate with the gut inflammatory status [14,53,54]. Increased levels of S100B protein are known in biopsies from UC-patients [54] and *in vitro* upon inflammatory stimulation. Mechanistically, secreted S100B induces an increase in glial inducible nitric oxide synthase (iNOS) and thereby leads to elevated secretion of nitric oxide (NO), a prominent pro-inflammatory mediator [11]. At first, our observations *in vivo* and the lack of *S100b* upregulation in the LPS stimulated gliospheres *in vitro* seem to be in conflict with the data obtained by Cirillo *et al*. However, a possible explanation lies in the nature of inflammatory stimulus: Cirillo *et al*. incubated tissue biopsies [14] and purified glial cultures [11] with a combination of LPS and interferon-gamma (IFN-γ) and describe that neither LPS nor IFN-γ alone had an effect on iNOS expression level, implying also a lack of effect of LPS alone on S100B expression and secretion. It can be followed that the LPS-mediated hyperinflammation model we employed elicits an activation of glia regarding their GFAP-status but this activated glial phenotype does not necessarily correlate to glial alterations concerning S100B secretion observed in IBD.

These *in vivo* findings on *Gfap* upregulation do not imply whether it affected cells that had been GFAP+ before the stimulation. It is also possible that GFAP- cells were induced to express *Gfap* upon LPS-stimulation, given the significant plasticity of GFAP expression in individual EGCs *in vitro* and *in vivo* [5,19]. To focus on the inflammation-induced molecular alterations specifically in the GFAP-expressing glial subpopulation, a transgenic hGFAP-EGFP mouse strain and established protocols for free-floating murine neurosphere-like bodies [34] were adapted to generate primary, purified “gliospheres” from flow cytometry-sorted GFAP+ glia. The GFAP+ population accounted for 4% of the total LMMP digest and could be propagated to form gliospheres that showed strong immunostaining for S100B and the intermediate filament GFAP but not Vimentin after prolonged *in vitro* culture. Vimentin and GFAP are described to be developmentally regulated in reverse manner, in immature glial cells GFAP expression is low and Vimentin is strongly expressed, whereas mature glia express high levels of GFAP and reduced levels of Vimentin [7]. Due to the high plasticity of GFAP expression 40% of an initially purified GFP-positive culture lost strong GFP-positivity. Interestingly, gliospheres contained αSMA-expressing cells. Since gliospheres were propagated from an initially purified GFP-positive population, an overgrowing contamination of the culture with non-glial muscle cells to such an extent seems unlikely within 2 weeks *in vitro*. This finding might rather be explained by the dependence of the glial cell phenotype on cues from their native microenvironment and consequently dedifferentiation of enteric glia in culture [55]. It is documented that enteric glia lose morphological and molecular characteristics *ex vivo* [7] and acquire capabilities not observed *in vivo* such as a neurogenic potential, or giving rise to αSMA-positive muscle cells [16,56]. In line with this, multipotent nestin-positive neural crest cells isolated from postnatal murine intestines were found to express both αSMA and GFAP after 1 day in culture and can subsequently dedifferentiate to muscle cells [57].

In a next step, we analyzed the secretion of inflammatory mediators by LPS-stimulated gliospheres. To determine a suitable time frame for the LPS stimulation we followed studies on cytokine secretion by stimulated CNS astrocytes *in vitro*, which measured strong effects after 24–48 h of stimulation [58,59]. Further, studies on EGC had shown that EGC-activation by LPS and subsequent secretion of EGC-derived growth factors or cytokines peaks between 24–48 h [19,13]. Indeed, we found that the primary GFAP+-glia derived gliospheres secreted different inflammatory cytokines upon extended LPS-stimulation such as IL-1β, IL-6, MCP-1 and IFN-γ, which was also described by studies conducted on the overall glial population [19,26]. Knowledge on distinct, subpopulation-specific glial functions is scarce. Our dataset indicates that the well-known ability of EGCs to release inflammatory cytokines in response to exogenous stimuli can be exerted by those cells derived from the GFAP+ subpopulation.

To unravel general molecular pathways as response to acute inflammation we performed a high-throughput transcriptomic study. LPS-treated enteric gliospheres showed 70 significantly differently expressed genes, often associated with immune responses, a signature also observed in reactive astrocytes of the CNS. Thus, the gene product of Lcn-2, Lipocalin-2, the gene with the highest fold change increase in our dataset, plays a role in the morphological and functional fate of reactive astrocytes, e.g. by inducing GFAP expression [60]. The gene products of Ccl2 and Ccl5, also known as MCP-1 and RANTES, are described to be produced by β-amyloid peptide-challenged astrocytes and act as microglial and macrophage chemoattractants *in vitro* [61]. Interestingly, we observed no significant upregulation of *Gfap*, *S100b* or other glial markers such as glial cell line-derived neurotrophic factor (GDNF), potentially indicating that purified primary GFAP-positive cells do not further upregulate synthesis of GDNF and S100B and that expression of these factors is induced in GFAP-low expressing cells during cell activation. Taken together, these data indicate that GFAP-EGCs are reactive to the acute LPS-stimulus *in vitro* and acquire a phenotype reminiscent of reactive CNS astrocytes.

Finally, our meta-analysis added more information to the aspect of analogy between EGCs and astrocytes. EGCs freshly isolated from mice showed close resemblance to astrocytes. Interestingly, while LPS treatment led to a dramatic change in gene expression profiles of CNS astrocytes and microglia, EGCs transcriptomes shifted at a much smaller extend. This might have a biological background since EGCs could be less sensitive to LPS due to the physiological exposure to bacterial components in the gut. Another possible explanation lies in the shift in transcriptomic profiles, which we observed after prolonged *in vitro* culture. Thus, the expanded gliospheres showed a molecular drift to mesenchymal cell entities, which correlates with the immunopositivity of gliospheres for αSMA and is likely caused by transdifferentiation of EGCs [16,57,62] or a reconstitution of an initially negligible muscle cell contamination in the cell-sorted culture. Another interesting aspect is that Rao et al. recently described a transcriptomic resemblance of Proteolipid Protein 1 (PLP1)+ EGCs to peripheral Schwann cells and only limited similarity to astrocytes [63]. This difference might be due to the low co-expression of PLP1 and GFAP by EGCs between 35–54% [63] and indicate the diversity of glial subpopulations.

Taken together, our data indicate that EGCs are responsive to an inflammatory environment within a short time frame. Administering LPS intravenously leads to a specific response of myenteric EGCs, adding new information to previous studies focusing on mucosal glia activation after epithelial barrier breakdown. It will be interesting to investigate if the transcriptomic shift in favor of immunomodulatory capacities observed *in vitro* can be verified *in vivo* and if EGCs indeed can modulate the onset and progression of inflammation. In the future, this knowledge might help us to better understand inflammatory disease progression and eventually aid to novel therapeutic treatment options.

## Supporting Information

S1 Fig Neuronal cells are not affected 4 h after LPS injection. (A) Quantitative PCR analysis of neuronal marker expression in whole intestinal wall preparations from duodenal, ileal and colonic segments. No inflammation-induced alterations are observed in mRNA expression levels of the overall neuronal marker *Uchl1* (*PGP 9*.*5*) or cholinergic (*Chat*) and nitrinergic (*Nos1*) subpopulations. (B) Hu-positive neuronal nuclei (C) numbers in the myenteric plexus are neither significantly affected, we only observe a tendency for reduced numbers in ileal segments. Results (mean ± standard deviation) are expressed as fold change relative to sham group set to 1, as indicated by the horizontal line. n = 3–5, ^#^ p < 0.10, scale = 100 μm.
(TIF)

S1 Table qPCR primers employed in this study. (DOCX)

S2 Table Gene Ontology datasets employed in the meta-analysis. (DOCX)

S3 Table Nanostring nCounter Codeset probes. Probes were designed to target the same genes at similar transcript positions as the Illumina probes of the microarray.
(DOCX)

S4 Table Significantly differentially expressed genes in LPS-stimulated gliospheres compared to unstimulated controls. (DOCX)

S5 Table Significantly enriched GO-terms assigned to SDEG. GO-terms describe biological processes based on the enrichment of differentially expressed genes upon LPS-stimulation.
(DOCX)
