# Supplementary figures and images for: Activation of Myenteric Glia during Acute Inflammation In Vitro and In Vivo
Source: PLoS One. 2016 Mar 10;11(3):e0151335. doi: 10.1371/journal.pone.0151335 (PMC4786261; doi:10.1371/journal.pone.0151335)

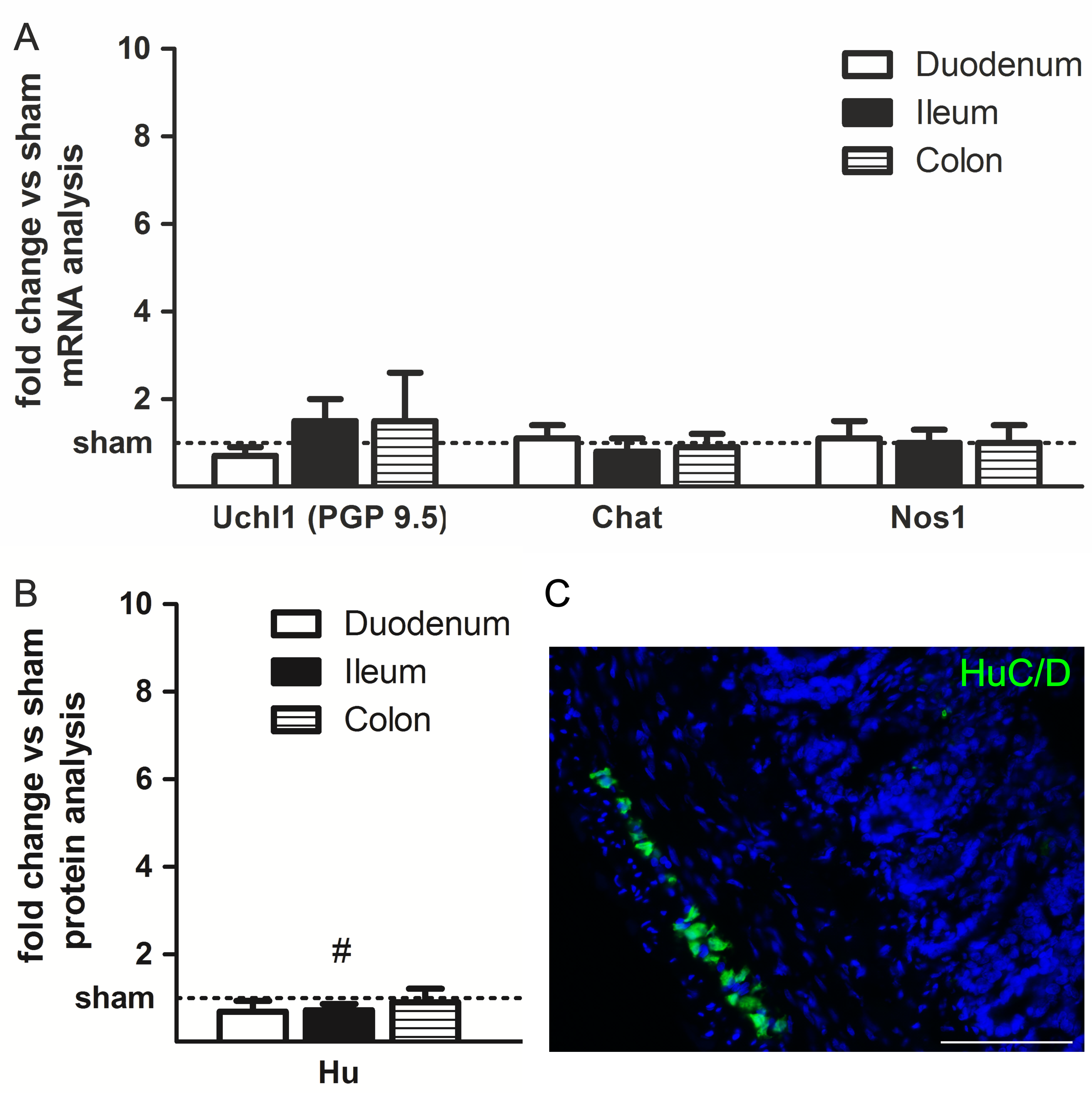

Supplement: S1 Fig — (A) Quantitative PCR analysis of neuronal marker expression in whole intestinal wall preparations from duodenal, ileal and colonic segments. No inflammation-induced alterations are observed in mRNA expression levels of the overall neuronal marker Uchl1 (PGP 9.5) or cholinergic (Chat) and nitrinergic (Nos1) subpopulations. (B) Hu-positive neuronal nuclei (C) numbers in the myenteric plexus are neither significantly affected, we only observe a tendency for reduced numbers in ileal segments. Results (mean ± standard deviation) are expressed as fold change relative to sham group set to 1, as indicated by the horizontal line. n = 3–5, # p < 0.10, scale = 100 μm. (TIF) [file pone.0151335.s001.tif]
