# Supplementary material for: Activation of Myenteric Glia during Acute Inflammation In Vitro and In Vivo
Source: PLoS One. 2016 Mar 10;11(3):e0151335. doi: 10.1371/journal.pone.0151335 (PMC4786261; doi:10.1371/journal.pone.0151335)
Supplement: S1 Table — (DOCX) [file pone.0151335.s002.docx]

**S1 Table. qPCR primers employed in this study.**

| **Gene** | **NCBI RefSeq** |  | **Primer sequence** | **size of product (bp)** |
| --- | --- | --- | --- | --- |
| *Gfap* | NM_017009.2 | sense | CCTGGAACAGCAAAACAAGGC | 203 |
|  |  | antisense | TTTCATCTTGGAGCTTCTGCCTC |  |
| *S100b* | NM_013191.1 | sense | TTCAGGGAGAGAGGGTGACAA | 104 |
|  |  | antisense | CTTCCTGCTCTTTGATTTCCTCC |  |
| *Uchl1 (PGP 9.5)* | NM_017237.2 | sense | TGAAGCAGACCATCGGGAAC | 178 |
|  |  | antisense | GCCTGAATGGCCTCGTTCTT |  |
| *Nos1* | NM_052799.1 | sense | AATCTCAGGTCGGCCATCAC | 126 |
|  |  | antisense | ATCCCCCAAGGTAGAGCCAT |  |
| *Chat* | NM_001170593 | sense | AAATGGCGTCCAACGAGGAT | 114 |
|  |  | antisense | CCCGGTTGGTGGAGTCTTTT |  |
| *Sod2* | NM_017051.2 | sense | CCCAAAGGAGAGTTGCTGGAG | 144 |
|  |  | antisense | CTGTAAGCGACCTTGCTCCT |  |
| *Actb* | NM_031144 | sense | GCAGGAGTACGATGAGTCCG | 74 |
|  |  | antisense | ACGCAGCTCAGTAACAGTCC |  |
| *18S rRNA* | M11188 | sense | GGGAGGTAGTGACGAAAAATAACAAT | 101 |
|  |  | antisense | TTGCCCTCCAATGGATCCT |  |
