# Supplementary material for: Activation of Myenteric Glia during Acute Inflammation In Vitro and In Vivo
Source: PLoS One. 2016 Mar 10;11(3):e0151335. doi: 10.1371/journal.pone.0151335 (PMC4786261; doi:10.1371/journal.pone.0151335)
Supplement: S2 Table — (DOCX) [file pone.0151335.s003.docx]

**S2 Table. Gene Ontology datasets employed in the meta-analysis.**

| **data set** | **Array Type** | **Array Manufacturer** | **Batch / GSE** | **Stimulation** |
| --- | --- | --- | --- | --- |
| bladder | moex10stv1 | Affymetrix | GSE15998 |  |
| BM derived neural crest cells | mouse430v2 | Affymetrix | GSE30419 |  |
| cerebral cortex | moex10stv1 | Affymetrix | GSE15998 |  |
| dorsal root ganglia | moex10stv1 | Affymetrix | GSE15998 |  |
| embryonic and induced pluripotent stem cells | mouseRef8v2 | Illumina | GSE36484 |  |
| embryonic and induced pluripotent stem cells | mouse430v2 | Affymetrix | GSE10806 |  |
| embryonic and induced pluripotent stem cells | mouse430v2 | Affymetrix | GSE10806 |  |
| embryonic and induced pluripotent stem cells | moex10stv1 | Affymetrix | GSE36017 |  |
| endothelial progenitor cells | mouse430v2 | Affymetrix | GSE29759 |  |
| epidermis | moex10stv1 | Affymetrix | GSE15998 |  |
| GFAP-EGC-P7 in vitro | mouseRef8v2 | Illumina | GSE78015 |  |
| GFAP-EGC-P7 in vitro +LPS | mouseRef8v2 | Illumina | GSE78015 |  |
| GFAP-EGC-P7 in vivo | mouseRef8v2 | Illumina | GSE78015 |  |
| gut | moex10stv1 | Affymetrix | GSE15998 |  |
| gut | moex10stv1 | Affymetrix | GSE15998 |  |
| heart | mouse430v2 | Affymetrix | GSE7196 |  |
| heart | moex10stv1 | Affymetrix | GSE15998 |  |
| hippocampus | moex10stv1 | Affymetrix | GSE15998 |  |
| kidney | moex10stv1 | Affymetrix | GSE15998 |  |
| liver | moex10stv1 | Affymetrix | GSE15998 |  |
| lung | moex10stv1 | Affymetrix | GSE15998 |  |
| MEF | mouse430v2 | Affymetrix | GSE8024 |  |
| MEF | moex10stv1 | Affymetrix | GSE26205 |  |
| MEF | moex10stv1 | Affymetrix | GSE36017 |  |
| MEF | mouseRef8v2 | Illumina | GSE30500 |  |
| MEF | mouseRef8v2 | Illumina | GSE21516 |  |
| MEF | mouseRef8v2 | Illumina | GSE36484 |  |
| mesenchymal stromal cells | mouse430v2 | Affymetrix | GSE30419 |  |
| mesenchymal stromal cells | mouseRef8v2 | Illumina | GSE31738 |  |
| NCSC | mouse430v2 | Affymetrix | GSE11149 |  |
| neural progenitor cells | mouseRef8v2 | Illumina | GSE36484 |  |
| neural progenitor cells | mouse430v2 | Affymetrix | GSE8024 |  |
| neural progenitor cells | mouse430v2 | Affymetrix | GSE10806 |  |
| neural progenitor cells | mouse430v2 | Affymetrix | GSE29759 |  |
| neural progenitor cells | mouseRef8v2 | Illumina | GSE30500 |  |
| olfactory bulb | moex10stv1 | Affymetrix | GSE15998 |  |
| ovary | moex10stv1 | Affymetrix | GSE15998 |  |
| placenta | moex10stv1 | Affymetrix | GSE15998 |  |
| prostate | moex10stv1 | Affymetrix | GSE15998 |  |
| sceletal muscle | moex10stv1 | Affymetrix | GSE15998 |  |
| spinal cord | moex10stv1 | Affymetrix | GSE15998 |  |
| stomach | moex10stv1 | Affymetrix | GSE15998 |  |
| umbilical cord | moex10stv1 | Affymetrix | GSE15998 |  |
| uterus | moex10stv1 | Affymetrix | GSE15998 |  |
| astrocytes | mouseRef8v2 | Illumina | GSE43808 | - |
| astrocytes +LPS | mouseRef8v2 | Illumina | GSE36089 | + LPS |
| GFAP-EGCs-P7 in vivo | mouseRef8v2 | Illumina | GSE78015 | - |
| GFAP-EGCs-P7 in vitro | mouseRef8v2 | Illumina | GSE78015 | - |
| GFAP-EGCs-P7 in vitro +LPS | mouseRef8v2 | Illumina | GSE78015 | + LPS |
| microglia 2 | mogene1.0_st | Affymetrix | GSE49329 | - |
| microglia 2 +LPS | mogene1.0_st | Affymetrix | GSE49329 | + LPS |
| microglia 1 | mouseRef8v2 | Illumina | GSE43808 | - |
| sciatic nerve, adult | mouseRef8v2 | Illumina | GSE44605 | - |
| sciatic nerve | mouseRef8v2 | Illumina | GSE16741 | - |
