# Supplementary material for: Activation of Myenteric Glia during Acute Inflammation In Vitro and In Vivo
Source: PLoS One. 2016 Mar 10;11(3):e0151335. doi: 10.1371/journal.pone.0151335 (PMC4786261; doi:10.1371/journal.pone.0151335)
Supplement: S3 Table — Probes were designed to target the same genes at similar transcript positions as the Illumina probes of the microarray. (DOCX) [file pone.0151335.s004.docx]

**S3 Table. Nanostring nCounter Codeset probes**. Probes were designed to target the same genes at similar transcript positions as the Illumina probes of the microarray.

| **Gene** | **Accession** | **Illumina ID** | **Illumina probe position** | **NanoString ID** | **design remarks** |
| --- | --- | --- | --- | --- | --- |
| Dag1 | NM_010017.4 | ILMN_2724585 | 4307-4406 | NM_010017.4:4306 |  |
| *Gfap* | NM_010277.3 | ILMN_1214715 | 1268-1367 | NM_010277.3:1267 |  |
| Hdac11 | NM_144919.2 | ILMN_2614912 | 2222-2321 | NM_144919.2:2221 |  |
| Hey2 | NM_013904.1 | ILMN_2657207 | 1853-1952 | NM_013904.1:1852 |  |
| Lama2 | NM_008481.2 | ILMN_2769479 | 8745-8844 | NM_008481.2:8744 |  |
| S100b | NM_009115.3 | ILMN_2634742 | 151-250 | NM_009115.3:150 |  |
| Bmp4 | NM_007554.2 | ILMN_1215252 | 1501-1600 | NM_007554.2:1500 |  |
| Bmp7 | NM_007557.3 | ILMN_2589662 | 1659-1758 | NM_007557.3:1658 |  |
| Cd82 | NM_007656.5 | ILMN_2747196 | 1410-1509 | NM_007656.5:1409 |  |
| Glul | NM_008131.4 | ILMN_2644496 | 2613-2712 | NM_008131.4:2612 |  |
| Gpc1 | NM_016696.4 | ILMN_2635784 | 3069-3168 | NM_016696.4:3068 | ~300 bp 5' of Illumina probe |
| Hgf | NM_010427.5 | ILMN_2729117 | 2397-2496 | NM_010427.5:2396 |  |
| Hmga2 | NM_010441.2 | ILMN_2743802 | 3418-3517 | NM_010441.2:3417 |  |
| Il5 | NM_010558.1 | ILMN_2731160 | 1300-1399 | NM_010558.1:1299 |  |
| Nog | NM_008711.2 | ILMN_2746870 | 1149-1248 | NM_008711.2:1148 |  |
| Plp1 | NM_011123.3 | ILMN_1240381 | 2432-2531 | NM_011123.3:2431 |  |
| Reln | NM_011261.2 | ILMN_2704257 | 11546-11645 | NM_011261.2:11545 |  |
| Sox5 | NM_011444.3 | ILMN_1213910 | 2553-2652 | NM_011444.3:2552 |  |
| Tnc | NM_011607.3 | ILMN_2485085 | 4883-4982 | NM_011607.3:4882 |  |
| Fgf5 | NM_010203.5 | ILMN_2642743 | 2179-2278 | NM_010203.5:2178 | ~5 bp 5' of Illumina probe |
| Cebpb | NM_009883.3 | ILMN_2756435 | 1148-1247 | NM_009883.3:1147 |  |
| Cxcl9 | NM_008599.4 | ILMN_1215862 | 921-1020 | NM_008599.4:920 |  |
| Nfkb1 | NM_008689.2 | ILMN_2592476 | 3470-3569 | NM_008689.2:3469 |  |
| Ripk2 | NM_138952.3 | ILMN_2634970 | 1541-1640 | NM_138952.3:1540 |  |
| Adam17 | NM_009615.6 | ILMN_2594718 | 4195-4294 | NM_009615.6:4194 |  |
| Ccl11 | NM_011330.3 | ILMN_2647757 | 404-503 | NM_011330.3:403 |  |
| Il13ra2 | NM_008356.3 | ILMN_1246284 | 1296-1395 | NM_008356.3:1295 |  |
| Irf5 | NM_012057.3 | ILMN_2621752 | 1827-1926 | NM_012057.3:1826 |  |
| Lcn2 | NM_008491.1 | ILMN_2712075 | 420-519 | NM_008491.1:419 |  |
| Mmp2 | NM_008610.2 | ILMN_2678218 | 2666-2765 | NM_008610.2:2665 | ~50 bp 5' of Illumina probe |
| Socs3 | NM_007707.3 | ILMN_2618176 | 2376-2475 | NM_007707.3:2375 | ~100 bp 5' of Illumina probe |
| Chil1 | NM_007695.3 | ILMN_2609813 | 1520-1619 | NM_007695.3:1519 |  |
| Cdk5 | NM_007668.3 | ILMN_1216721 | 1736-1835 | NM_007668.3:1735 |  |
| Id2 | NM_010496.3 | ILMN_1228557 | 625-724 | NM_010496.3:624 |  |
| Il6st | NM_010560.3 | ILMN_2608184 | 5075-5174 | NM_010560.3:5074 |  |
| Olig1 | NM_016968.4 | ILMN_2760105 | 1909-2008 | NM_016968.4:1908 | ~70 bp 5' of Illumina probe |
| Pou3f1 | NM_011141.2 | ILMN_1233172 | 1386-1485 | NM_011141.2:1385 |  |
| Rela | NM_009045.4 | ILMN_2740859 | 2610-2709 | NM_009045.4:2609 |  |
| Camp | NM_009921.2 | ILMN_2766604 | 376-475 | NM_009921.2:375 |  |
| Ccl2 | NM_011333.3 | ILMN_1245710 | 199-298 | NM_011333.3:198 |  |
| Ccl5 | NM_013653.3 | ILMN_1231814 | 295-394 | NM_013653.3:294 |  |
| Cd40 | NM_170702.2 | ILMN_2502136 | 769-868 | NM_170702.2:768 |  |
| Csf1 | NM_007778.4 | ILMN_1254561 | 3970-4069 | NM_007778.4:3969 |  |
| Csf2 | NM_009969.4 | ILMN_2749412 | 798-897 | NM_009969.4:797 |  |
| Il6 | NM_031168.1 | ILMN_1243601 | 647-746 | NM_031168.1:646 | ~200 bp 5' of Illumina probe |
| Mmp9 | NM_013599.3 | ILMN_2711075 | 2986-3085 | NM_013599.3:2985 |  |
| Ncam1 | NM_010875.3 | ILMN_2722864 | 2434-2533 | NM_010875.3:2433 |  |
| Tlr2 | NM_011905.3 | ILMN_2733733 | 2376-2475 | NM_011905.3:2375 |  |
| Agt | NM_007428.3 | ILMN_1227398 | 1175-1274 | NM_007428.3:1174 |  |
| Cxcl16 | NM_023158.6 | ILMN_2687586 | 1240-1339 | NM_023158.6:1239 |  |
| Fas | NM_007987.2 | ILMN_2479290 | 419-518 | NM_007987.2:418 |  |
| Ptn | NM_008973.2 | ILMN_2638114 | 1783-1882 | NM_008973.2:1782 |  |
| Runx1 | NM_009821.3 | ILMN_2589107 | 4143-4242 | NM_009821.3:4142 |  |
| Nkiras1 | NM_023526.3 | ILMN_2649696 | 1030-1129 | NM_023526.3:1029 | reference gene |
| B3gat1 | NM_029792.1 | ILMN_2724433 | 1552-1651 | NM_029792.1:1551 | reference gene |
| Dctn5 | NM_021608.3 | ILMN_2732536 | 1220-1319 | NM_021608.3:1219 | reference gene |
| Idh3b | NM_130884.4 | ILMN_1235050 | 1296-1395 | NM_130884.4:1295 | reference gene |
| Neu3 | NM_016720.2 | ILMN_2713763 | 2330-2429 | NM_016720.2:2329 | reference gene |
| Nupl1 | NM_170591.1 | ILMN_1245500 | 1439-1538 | NM_170591.1:1438 | reference gene |
| Pdha1 | NM_008810.2 | ILMN_2679851 | 2280-2379 | NM_008810.2:2279 | reference gene |
