# Supplementary material for: Activation of Myenteric Glia during Acute Inflammation In Vitro and In Vivo
Source: PLoS One. 2016 Mar 10;11(3):e0151335. doi: 10.1371/journal.pone.0151335 (PMC4786261; doi:10.1371/journal.pone.0151335)
Supplement: S4 Table — (DOCX) [file pone.0151335.s005.docx]

**S4 Table. Significantly differentially expressed genes in LPS-stimulated gliospheres compared to unstimulated controls**.

|  |  |  |  |  |  |  |
| --- | --- | --- | --- | --- | --- | --- |
| **Gene** | **Illumina ID** | **LogFC** | **Average expression** | **p value** | **Full gene name** | **Gene ontology term** |
| Ccl5 | ILMN_1231814 | 2,48 | 7,41 | 4,2E-10 | chemokine (C-C motif) ligand 5 | immune response |
| Mmp9 | ILMN_2711075 | 2,50 | 8,00 | 8,1E-10 | matrix metallopeptidase 9 | organic substance metabolic process |
| Ripk2 | ILMN_2634970 | 1,31 | 7,89 | 1,2E-09 | receptor (TNFRSF)-interacting serine-threonine kinase 2 | cellular response to stimulus |
| Cx3cl1 | ILMN_2627041 | 2,36 | 7,95 | 1,4E-09 | chemokine (C-X3-C motif) ligand 1 | immune response |
| Vmn1r181 | ILMN_2453946 | 2,43 | 7,99 | 1,7E-09 | vomeronasal 1 receptor 181 |  |
| Tnfaip2 | ILMN_2474858 | 1,69 | 7,52 | 1,0E-08 | tumor necrosis factor, alpha-induced protein 2 | single-organism cellular process |
| Cebpb | ILMN_2756435 | 2,58 | 10,59 | 1,1E-08 | CCAAT/enhancer binding protein (C/EBP), beta | biosynthetic process |
| Ms4a6d | ILMN_1241695 | 1,48 | 7,16 | 1,5E-08 | membrane-spanning 4-domains, subfamily A, member 6D |  |
| Ccl2 | ILMN_1245710 | 3,62 | 8,38 | 2,5E-08 | chemokine (C-C motif) ligand 2 | immune response |
| Tlr2 | ILMN_2733733 | 2,02 | 8,11 | 2,7E-08 | toll-like receptor 2 | cellular response to stimulus |
| Tnfaip3 | ILMN_1252202 | 1,51 | 7,61 | 4,3E-08 | tumor necrosis factor, alpha-induced protein 3 | cellular response to stimulus |
| Cxcl16 | ILMN_2687586 | 1,86 | 7,49 | 8,8E-08 | chemokine (C-X-C motif) ligand 16 | cellular response to stimulus |
| Csf2 | ILMN_2749412 | 1,24 | 7,06 | 1,0E-07 | colony stimulating factor 2 (granulocyte-macrophage) | immune response |
| Bcl3 | ILMN_2749717 | 1,42 | 7,70 | 1,1E-07 | B cell leukemia/lymphoma 3 | cellular metabolic process |
| Dcn | ILMN_2596346 | 2,73 | 8,82 | 1,6E-07 | decorin | cellular metabolic process |
| Gbp5 | ILMN_1244866 | 0,92 | 7,05 | 2,0E-07 | guanylate binding protein 5 | immune response |
| Nxn | ILMN_2677472 | 1,29 | 12,08 | 9,3E-07 | nucleoredoxin | single-organism cellular process |
| Pdlim4 | ILMN_2606162 | 2,38 | 10,39 | 1,2E-06 | PDZ and LIM domain 4 | single-organism cellular process |
| Rgs8 | ILMN_2732430 | 2,13 | 7,76 | 1,6E-06 | regulator of G-protein signaling 8 | cellular response to stimulus |
| Sox7 | ILMN_2653251 | 1,53 | 7,31 | 1,6E-06 | SRY-box containing gene 7 | cellular metabolic process |
| Nfkbie | ILMN_2690551 | 0,99 | 7,67 | 1,7E-06 | nuclear factor of kappa light polypeptide gene enhancer in B cells inhibitor, epsilon | biosynthetic process |
| Fcgr2b | ILMN_1228657 | 1,62 | 7,98 | 2,1E-06 | Fc receptor, IgG, low affinity IIb | immune response |
| Car13 | ILMN_1249727 | 1,60 | 7,67 | 2,5E-06 | carbonic anhydrase 13 | single-organism cellular process |
| Casp4 | ILMN_1254655 | 1,81 | 8,15 | 3,6E-06 | caspase 4, apoptosis-related cysteine peptidase | death |
| P2ry6 | ILMN_2663130 | 1,29 | 10,80 | 4,1E-06 | pyrimidinergic receptor P2Y, G-protein coupled, 6 | cellular response to stimulus |
| Slc11a2 | ILMN_2665063 | 0,83 | 8,33 | 6,2E-06 | solute carrier family 11 (proton-coupled divalent metal ion transporters), member 2 | establishment of localization |
| 4933426M11Rik | ILMN_1254551 | 1,32 | 10,99 | 9,2E-06 | RIKEN cDNA 4933426M11 gene |  |
| Sbno2 | ILMN_2638333 | 0,86 | 7,80 | 1,0E-05 | strawberry notch homolog 2 (Drosophila) | biosynthetic process |
| Gxylt2 | ILMN_2729015 | 0,93 | 7,24 | 1,1E-05 | glucoside xylosyltransferase 2 | cellular metabolic process |
| Lcn2 | ILMN_2712075 | 4,53 | 8,98 | 1,2E-05 | lipocalin 2 | establishment of localization |
| Mmp3 | ILMN_2704576 | 0,90 | 7,20 | 1,2E-05 | matrix metallopeptidase 3 | organic substance metabolic process |
| Gpr17 | ILMN_3160970 | -2,49 | 10,18 | 1,2E-05 | G protein-coupled receptor 17 | regulation of biological process |
| Crispld2 | ILMN_1257551 | 0,78 | 7,34 | 1,4E-05 | cysteine-rich secretory protein LCCL domain containing 2 | single organism cellular process |
| Trf | ILMN_2485323 | 1,02 | 11,04 | 1,4E-05 | transferrin | establishment of localization |
| Relb | ILMN_1246841 | 1,10 | 7,76 | 1,8E-05 | avian reticuloendotheliosis viral (v-rel) oncogene related B | immune response |
| Itga7 | ILMN_1257880 | 1,48 | 7,69 | 1,8E-05 | integrin alpha 7 | regulation of biological process |
| Mmp10 | ILMN_2619952 | 2,31 | 8,01 | 2,0E-05 | matrix metallopeptidase 10 | organic substance metabolic process |
| Cxcl5 | ILMN_1230278 | 1,89 | 7,37 | 2,1E-05 | chemokine (C-X-C motif) ligand 5 | immune response |
| Cd82 | ILMN_2747196 | 1,59 | 8,35 | 2,2E-05 | CD82 antigen |  |
| Cd14 | ILMN_2742075 | 1,46 | 7,51 | 2,3E-05 | CD14 antigen | immune response |
| Slc2a6 | ILMN_2618918 | 1,26 | 9,44 | 2,6E-05 | solute carrier family 2 (facilitated glucose transporter), member 6 | establishment of localization |
| Map3k8 | ILMN_1245924 | 1,17 | 7,35 | 2,8E-05 | mitogen-activated protein kinase kinase kinase 8 | regulation of biological process |
| Kcnn2 | ILMN_2595430 | -0,54 | 7,37 | 2,8E-05 | potassium intermediate/small conductance calcium-activated channel, subfamily N, member 2 | establishment of localization |
| Il13ra1 | ILMN_2668696 | 1,54 | 8,32 | 3,0E-05 | interleukin 13 receptor, alpha 1 | cellular response to stimulus |
| Nr1h3 | ILMN_2663374 | 0,84 | 7,19 | 3,7E-05 | nuclear receptor subfamily 1, group H, member 3 | biosynthetic process |
| Ramp1 | ILMN_2734391 | -1,51 | 9,09 | 4,0E-05 | receptor (calcitonin) activity modifying protein 1 | establishment of localization |
| Csf1 | ILMN_1254561 | 1,52 | 8,36 | 4,9E-05 | colony stimulating factor 1 (macrophage) | immune response |
| Saa3 | ILMN_2772632 | 4,17 | 8,83 | 5,0E-05 | serum amyloid A 3 | cellular response to stimulus |
| Ms4a6d | ILMN_2602352 | 0,63 | 7,05 | 6,1E-05 | membrane-spanning 4-domains, subfamily A, member 6D |  |
| Irf1 | ILMN_2599782 | 1,08 | 9,25 | 8,2E-05 | interferon regulatory factor 1 | immune response |
| 1110008P14Rik | ILMN_1225594 | 1,28 | 10,85 | 8,6E-05 | RIKEN cDNA 1110008P14 gene |  |
| Socs3 | ILMN_2618176 | 1,87 | 9,84 | 9,3E-05 | suppressor of cytokine signaling 3 | regulation of biological process |
| Fas | ILMN_2479290 | 1,58 | 7,80 | 9,8E-05 | Fas (TNF receptor superfamily member 6) | immune response |
| Col28a1 | ILMN_3161091 | -2,53 | 8,59 | 9,9E-05 | collagen, type XXVIII, alpha 1 | cellular metabolic process |
| Dram1 | ILMN_1219820 | 1,43 | 8,11 | 1,1E-04 | DNA-damage regulated autophagy modulator 1 | cellular metabolic process |
| Junb | ILMN_1220034 | 1,53 | 9,96 | 1,2E-04 | Jun-B oncogene | biosynthetic process |
| Nfkbiz | ILMN_2755008 | 2,18 | 9,34 | 1,2E-04 | nuclear factor of kappa light polypeptide gene enhancer in B cells inhibitor, zeta | biosynthetic process |
| Gadd45g | ILMN_2744890 | 1,24 | 8,32 | 1,3E-04 | growth arrest and DNA-damage-inducible 45 gamma | death |
| Mmp13 | ILMN_2737685 | 1,48 | 7,35 | 1,3E-04 | matrix metallopeptidase 13 | organic substance metabolic process |
| Ccrn4l | ILMN_1255422 | 1,23 | 8,99 | 1,3E-04 | CCR4 carbon catabolite repression 4-like (S. cerevisiae) | regulation of biological process |
| Chi3l1 | ILMN_2609813 | 2,44 | 8,13 | 1,3E-04 | chitinase 3-like 1 | single-organism cellular process |
| Casp8 | ILMN_1243876 | 0,73 | 8,31 | 1,4E-04 | caspase 8 | death |
| Hspb3 | ILMN_2748205 | -0,93 | 7,45 | 1,5E-04 | heat shock protein 3 | response to stress |
| Nrarp | ILMN_2596979 | -0,79 | 8,44 | 1,6E-04 | Notch-regulated ankyrin repeat protein | regulation of biological process |
| Cst3 | ILMN_1257575 | -1,10 | 13,51 | 1,6E-04 | cystatin C | response to stress |
| Cyp7b1 | ILMN_2601215 | 1,50 | 7,96 | 1,7E-04 | cytochrome P450, family 7, subfamily b, polypeptide 1 | organic substance metabolic process |
| Enpp1 | ILMN_1231851 | -1,15 | 8,81 | 1,8E-04 | ectonucleotide pyrophosphatase/phosphodiesterase 1 | immune response |
| Rassf10 | ILMN_2636005 | 0,84 | 7,31 | 2,1E-04 | Ras association (RalGDS/AF-6) domain family (N-terminal) member 10 | single-organism cellular process |
| Clu | ILMN_2727153 | 1,80 | 8,83 | 2,1E-04 | clusterin | death |
