# Supplementary material for: Activation of Myenteric Glia during Acute Inflammation In Vitro and In Vivo
Source: PLoS One. 2016 Mar 10;11(3):e0151335. doi: 10.1371/journal.pone.0151335 (PMC4786261; doi:10.1371/journal.pone.0151335)
Supplement: S5 Table — GO-terms describe biological processes based on the enrichment of differentially expressed genes upon LPS-stimulation. (DOCX) [file pone.0151335.s006.docx]

**S5 Table. Significantly enriched GO-terms assigned to SDEG.** GO-terms describe biological processes based on the enrichment of differentially expressed genes upon LPS-stimulation.

| **Biological process** | **Enrichment** | **p value Benjamini** | **Genes** |
| --- | --- | --- | --- |
| immune response | 7,7 | 2,6E-06 | Ccl5; Cx3cl1; Ccl2; Tlr2; Csf2; Bcl3; Gbp5; Fcgr2b; Sbno2; Relb; Cxcl5; Cd14; Fas; Gadd45g; Enpp1 |
| leukocyte activation | 9,8 | 9,9E-04 | Ripk2; Csf2; Bcl3; Fcgr2b; Sbno2; Relb; Irf1; Fas; Gadd45g |
| cell activation | 9,1 | 1,2E-03 | Ripk2; Csf2; Bcl3; Fcgr2b; Sbno2; Relb; Irf1; Fas; Gadd45g |
| defense response | 5,9 | 2,0E-03 | Ccl5; Ripk2; Ccl2; Tlr2; Bcl3; Fcgr2b; Trf; Cxcl5; Cd14; Saa3; Nfkbiz |
| leukocyte differentiation | 11,6 | 3,6E-03 | Csf2; Bcl3; Relb; Irf1;Fas; Gadd45g; Casp8 |
| regulation of cytokine production | 11,5 | 3,2E-03 | Ripk2; Cx3cl1; Cebpb; Tlr2; Bcl3; Cd14; Irf1 |
| positive regulation of multicellular organismal process | 10,7 | 4,3E-03 | Ripk2; Cx3cl1; Tlr2; Bcl3; Trf; Cd14; Csf1 |
| inflammatory response | 7,9 | 4,9E-03 | Ccl5; Ccl2; Tlr2; Trf; Cxcl5; Cd14; Saa3; Nfkbiz |
| hemopoiesis | 7,7 | 5,0E-03 | Csf2; Bcl3; Slc11a2; Relb; Irf1; Fas; Gadd45g; Casp8 |
| positive regulation of cytokine production | 20,7 | 6,3E-03 | Ripk2; Cx3cl1; Tlr2; Bcl3; Cd14 |
| response to wounding | 6,0 | 6,8E-03 | Ccl5; Cx3cl1; Ccl2; Tlr2; Trf; Cxcl5; Cd14; Saa3; Nfkbiz |
| collagen catabolic process | 40,3 | 6,8E-03 | Mmp9; Mmp3; Mmp10; Mmp13 |
| T cell activation | 12,1 | 6,6E-03 | Ripk2; Bcl3; Relb; Irf1; Fas; Gadd45g |
| hemopoietic or lymphoid organ development | 6,8 | 7,1E-03 | Csf2; Bcl3; Slc11a2; Relb; Irf1; Fas; Gadd45g; Casp8 |
| alpha-beta T cell differentiation | 38,0 | 6,6E-03 | Bcl3; Relb; Irf1; Gadd45g |
| multicellular organismal catabolic process | 38,0 | 6,6E-03 | Mmp9; Mmp3; Mmp10; Mmp13 |
| immune system development | 6,5 | 8,2E-03 | Csf2; Bcl3; Slc11a2; Relb; Irf1; Fas; Gadd45g; Casp8 |
| regulation of tumor necrosis factor production | 34,0 | 8,2E-03 | Ripk2; Tlr2; Bcl3; Cd14 |
| collagen metabolic process | 32,3 | 9,1E-03 | Mmp9; Mmp3; Mmp10; Mmp13 |
| alpha-beta T cell activation | 32,3 | 9,1E-03 | Bcl3; Relb; Irf1; Gadd45g |
| multicellular organismal macromolecule metabolic process | 32,3 | 9,1E-03 | Mmp9; Mmp3; Mmp10; Mmp13 |
| T cell differentiation | 15,2 | 1,1E-02 | Bcl3; Relb; Irf1; Fas; Gadd45g |
| multicellular organismal metabolic process | 29,3 | 1,1E-02 | Mmp9; Mmp3; Mmp10; Mmp13 |
| cell activation during immune response | 28,1 | 1,2E-02 | Bcl3; Sbno2; Relb; Gadd45g |
| leukocyte activation during immune response | 28,1 | 1,2E-02 | Bcl3; Sbno2; Relb; Gadd45g |
| T-helper cell differentiation | 96,8 | 1,2E-02 | Bcl3; Relb; Gadd45g |
| CD4-positive, alpha-beta T cell differentiation during immune response | 96,8 | 1,2E-02 | Bcl3; Relb; Gadd45g |
| response to lipoteichoic acid | 96,8 | 1,2E-02 | Ripk2; Tlr2; Cd14 |
| adaptive immune response | 14,2 | 1,2E-02 | Bcl3; Fcgr2b; Relb; Fas; Gadd45g |
| adaptive immune response based on somatic recombination of immune receptors built from immunoglobulin superfamily domains | 14,2 | 1,2E-02 | Bcl3; Fcgr2b; Relb; Fas; Gadd45g |
| myeloid leukocyte activation | 25,8 | 1,3E-02 | Csf2; Fcgr2b; Sbno2; Relb |
| T cell differentiation during immune response | 80,7 | 1,6E-02 | Bcl3; Relb; Gadd45g |
| alpha-beta T cell differentiation during immune response | 80,7 | 1,6E-02 | Bcl3; Relb; Gadd45g |
| regulation of apoptosis | 4,1 | 1,6E-02 | Mmp9; Ripk2; Cx3cl1; Cebpb; Csf2; Bcl3; Casp4; Fas; Casp8; Clu |
| regulation of programmed cell death | 4,0 | 1,7E-02 | Mmp9; Ripk2; Cx3cl1; Cebpb; Csf2; Bcl3; Casp4; Fas; Casp8; Clu |
| regulation of cell death | 4,0 | 1,8E-02 | Mmp9; Ripk2; Cx3cl1; Cebpb; Csf2; Bcl3; Casp4; Fas; Casp8; Clu |
| T-helper 1 type immune response | 69,2 | 1,9E-02 | Bcl3; Relb; Gadd45g |
| alpha-beta T cell activation during immune response | 69,2 | 1,9E-02 | Bcl3; Relb; Gadd45g |
| negative regulation of apoptosis | 6,2 | 1,9E-02 | Cx3cl1; Cebpb; Csf2; Bcl3; Casp4; Fas; Clu |
| negative regulation of programmed cell death | 6,1 | 2,0E-02 | Cx3cl1; Cebpb; Csf2; Bcl3; Casp4; Fas; Clu |
| negative regulation of cell death | 6,0 | 2,0E-02 | Cx3cl1; Cebpb; Csf2; Bcl3; Casp4; Fas; Clu |
| CD4-positive, alpha beta T cell differentiation | 60,5 | 2,2E-02 | Bcl3; Relb; Gadd45g |
| lymphocyte activation | 7,5 | 2,3E-02 | Ripk2; Bcl3; Relb; Irf1; Fas; Gadd45g |
| lymphocyte differentiation | 10,3 | 2,6E-02 | Bcl3; Relb; Irf1; Fas; Gadd45g |
| positive regulation of tumor necrosis factor production | 53,8 | 2,5E-02 | Ripk2; Tlr2; Cd14 |
| chemotaxis | 10,0 | 2,8E-02 | Ccl5; Cx3cl1; Ccl2; Cxcl16; Cxcl5 |
| taxis | 10,0 | 2,8E-02 | Ccl5; Cx3cl1; Ccl2; Cxcl16; Cxcl5 |
| T cell activation during immune response | 40,3 | 4,3E-02 | Bcl3; Relb; Gadd45g |
